# Pyridoxine supplementation ameliorates SGPL1 R222Q variant sphingosine phosphate lyase insufficiency syndrome in mice

**DOI:** 10.1172/JCI206980

**Published:** 2026-09-24

**Authors:** Ranjha Khan, Maria L. Allende, Ehtesham Khalid, Joanna Y. Lee, Everett Stone, Max Rodnick-Smith, Audrey Izuhara, Vadym Buncha, Georgina Gyarmati, Janos Peti-Peterdi, Ranya Al-Khaledy, Jeffrey B. Hodgin, Gizachew Tassew, Babak Oskouian, Alina Chen, Yu-Tang Chang, Rachel Zhang, Richard L. Proia, Julie D. Saba

**Affiliations:** 1 Department of Pediatrics, UCSF School of Medicine, San Francisco, California, USA.; 2 Genetics and Biochemistry Branch, National Institute of Diabetes and Digestive and Kidney Diseases, NIH, Bethesda, Maryland, USA.; 3 Ochsner Clinical School/University of Queensland (Australia) and Ochsner Health, New Orleans, Louisiana, USA.; 4 Department of Molecular Biosciences, University of Texas, Austin, Texas, USA.; 5 Department of Oncology, University of Texas Dell Medical School, LIVESTRONG Cancer Institutes, Austin, Texas, USA.; 6 Department of Physiology and Neuroscience and Department of Medicine, Zilkha Neurogenetic Institute, University of Southern California, Los Angeles, California, USA.; 7 Department of Pathology, University of Michigan School of Medicine, Ann Arbor, Michigan, USA.

**Keywords:** Metabolism, Nephrology, Monogenic diseases, Mouse models, Therapeutics

## Abstract

Sphingosine-1-phosphate lyase insufficiency syndrome (SPLIS) is a rare condition causing nephrotic syndrome, neuropathy, and other manifestations. SPLIS is caused by mutations in *SGPL1*, which encodes sphingosine-1-phosphate lyase (SPL), a pyridoxal 5′-phosphate–dependent (PLP-dependent) enzyme needed to degrade the bioactive sphingolipid sphingosine-1-phosphate (S1P). Supplementation with the PLP precursor pyridoxine benefits some individuals with PLP-dependent enzymopathies. We investigated whether pyridoxine has therapeutic activity in SPLIS. Neurological improvement, plasma S1P normalization, and increased SPL activity in patient-derived fibroblasts were observed after pyridoxine supplementation in a patient with R222Q-variant SPLIS. Additionally, PLP dose-dependently augmented recombinant R222Q-variant SPL activity. To further explore pyridoxine’s effects, gene editing was employed to create an R222Q-variant SPLIS mouse model. SPL^R222Q^ mice fed pyridoxine-enriched chow lacked obvious phenotypes. However, SPL inactivation, S1P accumulation, proteinuria, and glomerulosclerosis developed in SPL^R222Q^ but not WT mice fed chow with reduced pyridoxine. Ultrastructural analysis and super-resolution microscopy showed podocyte loss and foot process effacement. Transcriptional profiling revealed patterns of cytokine upregulation and extracellular matrix remodeling. Inhibiting S1P production or RhoA/ROCK signaling prevented nephrosis in SPL^R222Q^ mice fed chow lacking pyridoxine. Our findings establish a SPLIS mouse model that recapitulates R222Q-variant SPLIS, demonstrates its responsiveness to pyridoxine, and implicates a S1P/RhoA/ROCK pathway in its pathophysiology.

## Graphical abstract

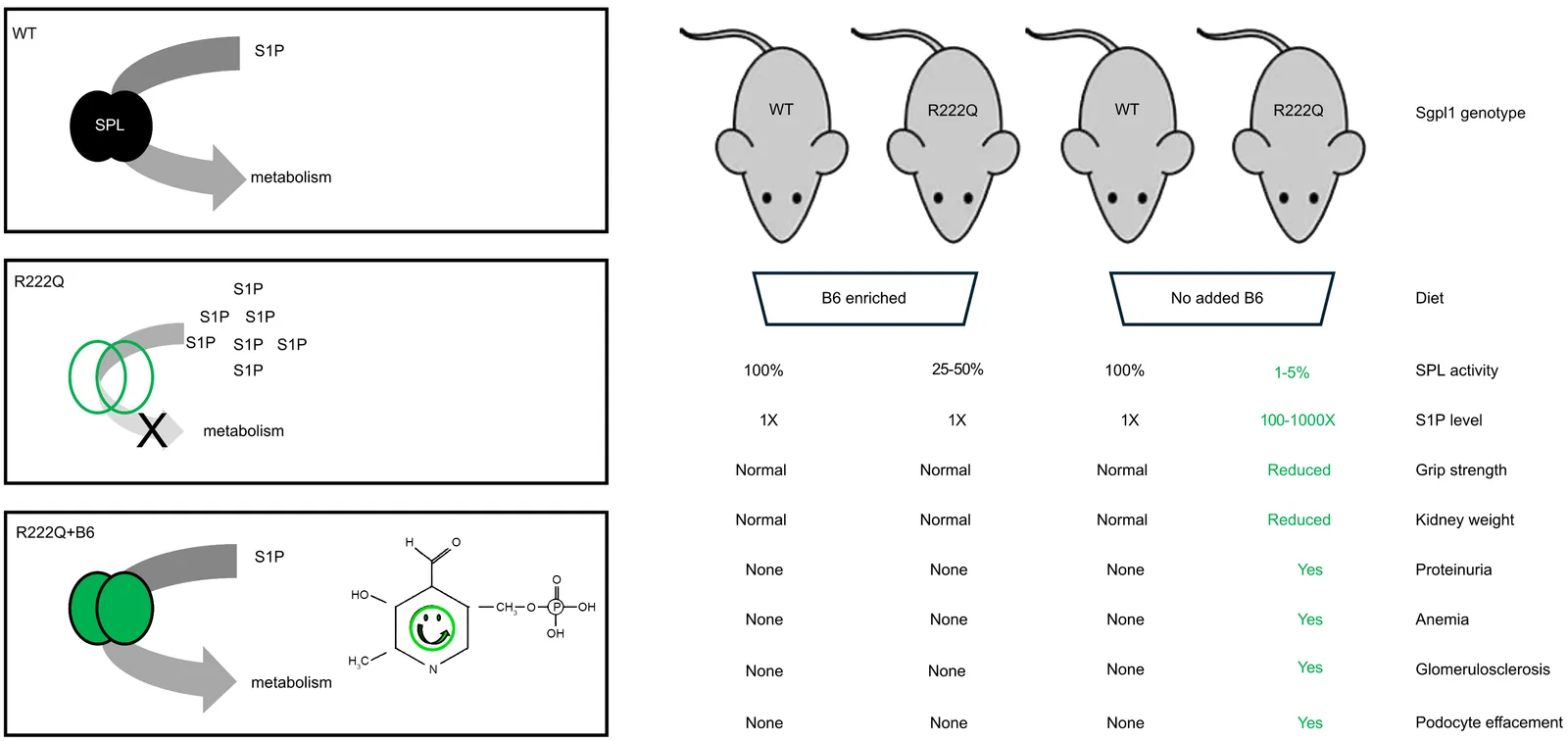

## Introduction

Sphingosine phosphate lyase insufficiency syndrome (SPLIS), also known as nephrotic syndrome type 14 (OMIM #617575), is an inborn error of sphingolipid metabolism (1, 2). SPLIS has an estimated prevalence of 11,000 worldwide (3). Its main features include steroid-resistant nephrotic syndrome, adrenal insufficiency, peripheral neuropathy, anemia, and immunodeficiency. When diagnosed in infancy with kidney involvement, SPLIS has an 80% mortality within years (4). Currently, there is no targeted therapy for SPLIS.

SPLIS is caused by biallelic inactivating *SGPL1* mutations (OMIM #603729; HGNC: 10817). Over 45 SPLIS-associated *SGPL1* variants are known, the most common being the R222Q substitution, accounting for approximately 20% of SPLIS cases (5). *SGPL1* encodes sphingosine-1-phosphate lyase (SPL), an essential, pyridoxal 5′-phosphate–dependent (PLP-dependent) enzyme that catalyzes the irreversible cleavage of the bioactive lipid sphingosine-1-phosphate (S1P) in the last step of sphingolipid metabolism (6, 7). Complex biochemical consequences of SPL insufficiency contribute to SPLIS pathogenesis. Inability to degrade S1P results in S1P accumulation in cells, blood, and tissues (8). This may induce aberrant S1P signaling through S1P receptors, which regulate vascular permeability, leukocyte trafficking, neurotransmission, and proinflammatory and profibrotic cytokine signaling (9). Receptor-independent effects of S1P on calcium homeostasis, epigenetic gene transcription, cholesterol handling, and mitochondrial function may additionally occur in SPLIS (10, 11). All sphingolipids must eventually be metabolized through the common sphingolipid degradative pathway ending in SPL. Consequently, SPL inactivation causes accumulation of upstream sphingolipid metabolites, such as ceramides, which can be cytotoxic, and deficiency of the metabolic products, hexadecenal and ethanolamine phosphate, which contribute to autophagy and phospholipid biosynthesis (12–16). An ideal SPLIS treatment would address all the biochemical consequences of SPL insufficiency. This could be accomplished by augmenting endogenous SPL activity or providing a working copy of the *SGPL1* gene or SPL protein, thereby restoring sphingolipid metabolism to key tissues.

PLP is the active form of vitamin B6 and 1 of 6 B6 vitamers, the other 5 being enzymatically convertible to PLP in vivo (17). Clinical guidelines for infantile seizures began recommending use of the B6 vitamer pyridoxine in the 1980s, and its use in pediatrics is well established. Pyridoxine also has therapeutic activity in some inborn errors of metabolism involving PLP-dependent enzymes (18). By increasing intracellular PLP concentrations, pyridoxine supplementation may overcome the poor cofactor-binding affinity of some pathogenic enzyme variants. Alternatively, PLP may act as a chaperone, stabilizing variants prone to misfolding (19). These 2 mechanisms are not mutually exclusive.

We hypothesized that pyridoxine supplementation would benefit a subset of SPLIS patients harboring pyridoxine-responsive SPL variants. Pyridoxine supplementation led to clinical and biochemical improvements in 2 SPLIS patients, including 1 homozygous for the SPL R222Q variant (20). Given pyridoxine’s favorable safety profile and widespread availability, establishing the efficacy of pyridoxine supplementation in SPLIS (or a tractable model of SPLIS) and identifying pyridoxine-responsive SPL variants are clinically valuable goals. However, SPLIS’s rarity and genetic and phenotypic heterogeneity as well as the difficulty of convincing patients to participate in an interventional study when pyridoxine is freely available make conducting a pyridoxine randomized clinical trial in SPLIS challenging.

Here, we sought to determine whether pyridoxine supplementation can prevent disease progression and reverse organ damage in SPLIS using preclinical models including patient-derived skin fibroblasts, purified recombinant SPL protein, and a personalized mouse model of R222Q-variant SPLIS. Our results establish SPL^R222Q^ mice as a unique, inducible SPLIS model that exhibits SPLIS phenotypes when B6 is limiting but not under conditions of pyridoxine enrichment. Our results demonstrate pyridoxine’s efficacy in the treatment of SPLIS caused by the most common pathogenic SPL variant. Furthermore, preventing S1P production in vivo using sphingosine kinase inhibitors or downstream activation of the RhoA/ROCK pathway using fasudil prevents development of SPLIS nephrosis, implicating S1P accumulation and downstream RhoA/ROCK signaling as a culprit in SPLIS pathophysiology.

## Results

### Pyridoxine attenuates disease manifestations in a patient with SPL R222Q-variant SPLIS.

An 18-year-old female patient presented with peripheral numbness, burning sensation, and extremity pain beginning at age 8 years. Family history was notable for parental consanguinity, 2 miscarriages, and death of 2 female siblings. One had delayed milestones, skin hyperpigmentation, and died at age 2 and a half due to a fever. The other died at age 2 with kidney problems and proteinuria (Supplemental Figure 1A; supplemental material available online with this article; https://doi.org/10.1172/JCI206980DS1). Despite this, repeated measurements showed no proteinuria (Supplemental Table 1). The patient’s baseline nerve conduction study (NCS) suggested axonal polyneuropathy with no evidence of demyelination. Whole-exome sequencing (WES) of the proband identified a homozygous *SGPL1* R222Q pathogenic variant previously associated with SPLIS (Supplemental Figure 1B). Both parents were heterozygous for the variant. The substituted arginine is highly conserved in the eutherian mammals (Supplemental Figure 1C). We previously showed disease-modifying activity of cofactor supplementation using pyridoxine in 2 individuals with SPLIS, 1 of whom was homozygous for the R222Q variant (20). To assess whether pyridoxine might benefit the patient, SPL expression was measured by IB in extracts of patient-derived and healthy control skin fibroblasts cultured in pyridoxine-free medium. A strong SPL protein band was observed in control fibroblasts, but only a faint band was observed in the SPLIS fibroblasts, whereas SPLIS fibroblasts subsequently treated with 100 μM pyridoxine for 72 hours showed increased signal, indicating a stabilizing effect of cofactor supplementation (Supplemental Figure 1D). Correspondingly, baseline SPL enzyme activity in patient-derived fibroblasts was 5% of control levels, consistent with an inborn error of metabolism (Supplemental Figure 1E). Pyridoxine treatment resulted in an increase in SPL activity to 25% of control (Supplemental Figure 1E), a level that correlates with prevention of severe SPLIS phenotypes in murine SPLIS models (21). Based on this result, a trial of 100 mg/day oral pyridoxine was initiated. Over several months, the patient reported 80% improvement in neurological function, including improved strength and resolution of neuralgias. Median motor nerve conduction (Supplemental Figure 1F) and median and ulnar sensory nerves (data not shown) showed 50% improvement. Concomitantly, the patient’s plasma S1P levels, which were significantly elevated over age- and gender-matched control levels before pyridoxine treatment, fell into normal range with treatment (Supplemental Figure 1G).

### PLP treatment augments the catalytic activity of recombinant R222Q-variant SPL.

To assess whether PLP supplementation influences the activity of SPL variants, recombinant WT human SPL and SPLIS-associated R222Q and R222W variants were expressed in *E*. *coli* and purified in the presence of excess PLP. The affinity chromatography step yielded a prominent band corresponding to the expected molecular weight of human SPL (~55 kDa) (Figure 1A). The R222Q variant exhibited slightly reduced yield, whereas the R222W variant yield was profoundly reduced (Figure 1A). Recombinant SPL proteins copurified with contaminants that were eliminated with ion exchange chromatography (Figure 1A). To characterize the enzymatic activities of WT and variant SPL proteins, a time-course reaction was performed using 50 nM of the holoenzyme and 10 μM of S1P. The R222Q variant showed enhanced activity with PLP supplementation, while the R222W variant showed virtually no activity with or without PLP (Figure 1B). Similarly, we compared the activity of R222Q and R222W variants after normalizing to WT enzyme. Again, R222Q variant activity was augmented with increasing PLP, whereas R222W variant was catalytically dead. WT SPL activity was not improved by PLP supplementation (Figure 1C). Thus, PLP dose-dependently recovered the enzyme activity of the R222Q variant, whereas increasing PLP had little to no effect on WT enzyme or R222W variant.

### Generation of a murine model of R222Q-variant SPLIS.

Based on the above findings, it became important to validate pyridoxine’s prophylactic effects in vivo. A well-characterized *Sgpl1-*KO mouse is characterized by elevated blood and tissue sphingolipids, SPLIS-associated phenotypes, runting, and perinatal death (7, 22, 23). However, *Sgpl1*-KO mice produce no SPL protein, making this model unsuitable for testing pyridoxine’s efficacy in SPLIS. We generated mice harboring the SPL R222Q substitution using CRISPR genome editing technology (Figure 2A). A single founder female was identified who harbored 1 *Sgpl1* allele resulting in a gene product with the R222Q substitution. A SphI restriction cleavage site created by the nucleotide change allowed us to detect this variant by PCR followed by DNA digestion with SphI, resulting in 2 DNA fragments diagnostic of the knockin mutation (Figure 2B). Interestingly, an unintentional 38 bp *Sgpl1* deletion occurred in the *Sgpl1* gene on the founder’s other chromosome (Figure 2A). Both knockin and deletion mutations were verified by Sanger sequence analysis (Figure 2A). Unexpectedly, the founder mouse was healthy and reproductive (Figure 2C). When bred with a WT mate, she bore pups, some of which carried the R222Q variant. The heterozygous knockin pups were bred to homozygosity for the SPL R222Q variant, and homozygotes were used for further analysis.

### Pyridoxine supplementation augments tissue SPL activity and prevents S1P accumulation in SPL^R222Q^ mice.

We originally hypothesized that mice homozygous for the R222Q variant (hereafter referred to as SPL^R222Q^ mice) would exhibit SPLIS-like phenotypes that would be ameliorated by pyridoxine supplementation. However, the healthy state of the founder mouse, which harbored a deletion in 1 *Sgpl1* allele and relied on the R222Q variant for function, showed no obvious phenotype under standard husbandry conditions. Similarly, SPL^R222Q^ mice showed no phenotype under the same conditions. Maintenance pyridoxine requirements for mice are approximately 1 mg/kg/d (24). Based on the pyridoxine content of regular laboratory mouse chow and an estimated intake of 5 g chow/day, the mice were receiving approximately 6–7 mg/kg/d pyridoxine, i.e., 6- to 7-fold higher than minimum daily requirements. Thus, standard mouse chow contains supplementation with what could represent a “therapeutic” amount of pyridoxine. To determine the impact of eliminating pyridoxine supplementation on the R222Q variant, we compared SPL expression in vital organs of SPL^R222Q^ mice given standard chow (Hi-B6) versus chow measuring 2 ppm pyridoxine with no added pyridoxine (NA-B6) for 2 months (Supplemental Methods). To ensure we were not eliciting phenotypes caused by general B6 deficiency, we included groups of WT mice on Hi-B6 and NA-B6 chow for comparison. IB of whole kidney extracts revealed robust SPL protein expression in WT kidneys regardless of diet, indicating WT SPL abundance is unaffected when pyridoxine supplementation is withdrawn for 2 months (Figure 3A). This is consistent with the lack of effect of increasing amounts of PLP on purified WT SPL (Figure 1, B and C) and the fact that the WT SPL polypeptide folds into its native conformation, is abundant and stable, and exhibits a high affinity for PLP. In contrast, SPL protein levels were reduced in the kidneys of SPL^R222Q^ mice fed Hi-B6 chow, with even lower SPL levels observed in SPL^R222Q^ mice fed NA-B6 chow (Figure 3A). Consistent with the WT expression pattern, SPL activity levels in WT mouse liver, lung, kidney, and brain were comparable on either diet (Figure 3, B–E). SPL activity in tissues of SPL^R222Q^ mice fed Hi-B6 chow was 25%–50% of WT, a level shown to prevent most SPLIS-associated phenotypes in mice and consistent with the lack of reported disease phenotypes in SPLIS carriers (21). In contrast, SPL activity in liver, lung, and kidney of SPL^R222Q^ mice fed NA-B6 chow decreased to 1%–5% of the levels observed in tissues of WT mice on the same chow, a range comparable with that of an inborn error of metabolism, with somewhat more residual SPL activity observed in brain (Figure 3, B–E). S1P levels in plasma and tissues of WT and SPL^R222Q^ mice fed Hi-B6 chow and WT mice on NA-B6 chow were similar (Figure 3, F–J). This indicates that the residual global SPL activity in SPL^R222Q^ mice fed Hi-B6 chow is sufficient to maintain normal sphingolipid metabolism. In contrast, plasma and tissue S1P levels of SPL^R222Q^ mice fed NA-B6 chow were highly elevated compared with the control groups (Figure 3, F–J).

### Pyridoxine withdrawal induces development of SPLIS phenotypes in SPL^R222Q^ mice.

We next investigated whether dietary pyridoxine influences the development of SPLIS-related phenotypes in SPL^R222Q^ mice. Grip strength as a measure of neurological function (Figure 4A) and kidney weight (Figure 4B) deteriorated in SPL^R222Q^ mice after 2 months on NA-B6 chow, compared with all other groups. Urine albumin creatinine ratio (ACR), a measure of proteinuria, rose significantly in SPL^R222Q^ mice after 3 weeks on NA-B6 chow (Figure 4C) and increased further after 2 months (Figure 4D). Correspondingly, serum albumin was lower in SPL^R222Q^ mice after 2 months on NA-B6 chow compared with all other groups (Figure 4E). RBC indices were also lower, indicating development of anemia in SPL^R222Q^ mice after 2 months on the NA-B6 diet, compared with all other groups (Figure 4, F–H).

### Pyridoxine withdrawal induces glomerulosclerosis, podocyte loss, and foot process effacement in SPL^R222Q^ mice.

Histology of periodic acid Schiff–stained (PAS-stained) kidney sections of WT and SPL^R222Q^ mice on Hi-B6 chow and WT mice on NA-B6 chow was normal in appearance and indistinguishable from one another (Figure 5, A–C). In contrast, PAS staining of SPL^R222Q^ kidneys after 2 months on NA-B6 chow showed glomerulosclerosis with collagen deposition, mesangial expansion, and protein casts (Figure 5D). After 2 months on NA-B6 chow, SPL^R222^ mice exhibited 8% focal glomerulosclerosis and 13% global glomerulosclerosis (Supplemental Table 2). Injury and death of glomerular podocytes, which maintain the kidney filtration barrier, are considered the main underlying causes of steroid-resistant nephrotic syndrome (25). Podocyte foot process loss indicates podocyte injury and is pathognomonic of nephrotic syndrome. Ultrastructural analysis of kidney cortices of SPL^R222Q^ mice fed NA-B6 chow by transmission electron microscopy revealed podocyte foot process effacement and glomerular basement membrane thickening, features not observed in control kidneys (Figure 5, E and F). Stimulated emission depletion (STED) super-resolution microscopy of kidney cortices stained with antibodies against the podocyte marker nephrin demonstrated reduced slit diaphragm density (podocyte effacement) and overall reduced nephrin immunolabeling, indicating podocyte dropout in kidneys of SPL^R222Q^ mice on NA-B6 chow compared with controls (Figure 5, G–K).

### Pyridoxine withdrawal causes activation of stress signaling in SPL^R222Q^ mouse kidneys.

Expression of podocyte markers nephrin and synaptopodin was reduced in extracts of SPL^R222Q^ kidneys after withdrawal of pyridoxine supplementation, as shown by IB (Figure 6, A and B). To gain further insight into the molecular events leading to podocyte injury, we used IB of whole kidney extracts to compare the 4 groups of mice for the activity of pathways known to be activated by S1P signaling and/or SPL insufficiency and implicated in the pathogenesis of fibrosis, including AKT (26, 27), ERK (27–29), PKCδ (30), STAT3 (23), and SMAD3/4 (31). In each case, we observed upregulation of the phosphorylated (active) form of the signaling protein in SPL^R222Q^ mouse kidneys upon pyridoxine withdrawal, but not in the 3 control groups. STAT3 and S1P signaling pathways are mutually coactivating (32–34). STAT3 activation has been linked to diabetic kidney disease and other forms of glomerular injury (35–37). We performed p-STAT3 IHC and observed increased signals of p-STAT3, especially in the tubule cells and interstitium of SPL^R222Q^ mouse kidneys after withdrawal of pyridoxine supplementation, but not in control groups (Figure 7A). Moreover, qPCR confirmed that downstream targets of STAT3 transcriptional regulation (*Lcn2*, *Timp1*, *Socs1*, and *Socs3*) were upregulated in kidneys of SPL^R222Q^ mice after withdrawal of pyridoxine (Figure 7B).

### Pyridoxine withdrawal induces a transcriptional profile reflecting tissue injury and ECM remodeling in SPL^R222Q^ mouse kidneys.

To assess the impact of SPL insufficiency on kidney homeostasis, we conducted a transcriptome analysis using RNA-seq of whole kidneys from WT and SPL^R222Q^ mice on Hi-B6 chow and 3 weeks after switching to NA-B6 chow. Pearson’s correlation and principal component analysis revealed a distinct, significantly altered expression pattern of kidney transcripts in SPL^R222Q^ mice transferred to NA-B6 chow not observed in the other 3 groups (Supplemental Figure 2), depicted using heatmaps and the overall transcriptome profile (Supplemental Figures 3 and 4). We also identified genes differentially expressed in response to pyridoxine alone by comparing kidney transcriptomes of WT Hi-B6 versus WT NA-B6 groups. Not surprisingly, this led to identification of many B6-dependent genes, of which 9 were also altered in SPL^R222Q^ mice kidneys on NA-B6 chow when compared with WT on NA-B6. These genes included *Hdc*, encoding histidine decarboxylase; the cytochrome p450 genes *Cyp7b1* and *Cyp4a14*; *Spp1*, encoding osteopontin; the fibronectin gene *Fn1*; *Calb1*, encoding calbindin 1; *Pigr*, encoding the polymeric immunoglobulin receptor; *Chd9*, encoding chromodomain helicase DNA binding protein 7; and *Slc7a13*, encoding the cystine transporter. Of these, only histidine decarboxylase is known to functionally depend upon pyridoxal 5′-phosphate. The entire SPL-dependent gene set is shown in Figure 8A. Of note, kidney injury molecule 1 (*Kim1/Havcr1*), an established indicator of kidney injury, was upregulated in SPL^R222Q^ mice on NA-B6 chow (38). Gene Ontology (GO) enrichment analysis revealed other related processes enriched by SPL insufficiency. Pathways involved in the innate immune response, including chemokine- and cytokine-mediated signaling (*Osrm*, *Ccr5*, *Cx3Cr1*, *Cxcl15*, and *C3ar1*), TNF-α signaling (*Gadd45g*, *Fam129a*, and *Hsph1*), macrophage activation (*Cd68*), and complement activation (*Pzp*, *Hrg*, *Serpina16*, *Wfdc8*, *Spint4*, and *Tmprss11a*), were differentially expressed (all upregulated except *Cxcl15*), consistent with their roles in the pathogenesis of kidney injury and fibrosis (39–41). Upregulation of genes related to IL-17 (*Mmp2* and *Mmp14*), a STAT3 transcriptional target, is consistent with our findings of STAT3 activation in kidneys of SPL^R222Q^ mice on NA-B6 (42). An interconnected signaling network that includes interactions between TGF-β/SMAD, YAP/TAZ, IGF, and HIPPO pathways central to tissue repair and fibrosis (*Igfbp1*, *Grem2*, *Stc1*, *St2*, *Pappa*, *Axl*, and *Adamtsl2*) was differentially expressed (all upregulated except *Adamtsl2*) (43). Eleven genes representing collagen fibril organization and ECM remodeling pathways directly involved in fibrosis were dysregulated in SPL-deficient kidneys (*Col12a1*, *Col1a1*, *Col1a2*, *Dcn*, *Ddr2*, *Adamtsl2*, *Lamc3*, *Zfp469*, *Dsg3*, *MMP2*, and *MMP14*), all upregulated, except *Lamc3* and *adamtsl2*. This indicates that injury signals leading to maladaptive tissue repair were already present in kidneys after 3 weeks of low B6 conditions, before pathological signs of fibrosis could be detected (31, 44). In addition, cholesterol metabolism (*Apoa4*, *Apob*, and *Azgp1*) and lipid metabolic pathways that play roles in podocyte injury and kidney disease and interact closely with sphingolipids were upregulated (45–47). Protein degradation, known to be influenced by SPL through the endoplasmic reticulum stress response pathway and other mechanisms, was dysregulated in SPL-deficient kidney (13, 48). Response to infections with *Staphylococcus aureus*, *Influenza A*, *Entamoeba histolytica*, and *Plasmodium* were functionalities represented by genes corresponding with immune, ECM, and cytokine pathways mentioned above (Figure 8B). Six genes involved in vascular smooth muscle contraction (*Slc8a1*, *Gpr176*, *Scn2b*, *Clca3a1*, *Cacna1s*, and *Pvalb*) were upregulated, which may be explained by S1P’s vasoconstrictive effects on preglomerular microvessels (49). Genes involved in arginine and proline metabolism are upregulated, likely because of PLP’s role in converting arginine to proline and thus may not be relevant to SPL inactivation independent of pyridoxine status. Transcriptomic profiling of mutant mouse kidneys identified the chemokine signaling pathway as being prominently activated. Consistent with KEGG pathway analysis (Supplemental Figure 5), several major downstream effectors of chemokine signaling, including STAT3, ERK1, AKT, and Ca²^+^-associated signaling (PKC), were implicated in SPL^R222Q^ mice kidneys. These RNA-seq findings corroborated our IB results demonstrating activation of STAT3, ERK1, and AKT, along with enhanced calcium-related signaling. Taken together, these results indicate that chemokine signaling activation in mutant kidneys may drive renal inflammatory and downstream cellular responses. Using qPCR to measure gene expression, we confirmed upregulation of key transcripts, including established kidney injury markers (*Kim1/Havcr1* and *Pappa*), chemokines and their receptors (*Cxcr1*, *Ccl17*, *Ccl2/Mcp1*, and *Cxcl10*), and proinflammatory and profibrotic cytokines (*Tnfa* and *Tgfb*) (Figure 8C). In addition, we observed upregulation of *Tnfsf15*, a cytokine recently linked to nonmonogenic steroid-sensitive nephrotic syndrome (50). Altogether, kidney transcriptional profiling confirmed that a process of proinflammatory signaling and ECM remodeling, ultimately leading to glomerulosclerosis and podocyte injury, is induced within weeks after discontinuing pyridoxine supplementation in SPL^R222Q^ mice.

### SPLIS nephrosis is caused by S1P accumulation.

Development of SPLIS phenotypes in SPL^R222Q^ mice receiving limiting amounts of B6 coincided with a striking rise in circulating and tissue S1P levels, implicating S1P in the pathogenesis of SPLIS. This notion was further supported by upregulation of S1P-linked signaling pathways in their kidneys. However, the pathogenic role of SPL product deficiencies and/or accumulation of other sphingolipids could not be discounted. To test whether S1P accumulation mediates the SPLIS nephropathy, we placed SPL^R222Q^ mice on NA-B6 chow for 2 months, followed by 2 weeks of parenteral administration of SKI-II, a catalytic inhibitor of SphK1 and SphK2 that also induces proteolytic SphK1 degradation. Treating SPL^R222Q^ mice with SK-II rescued kidney barrier function and overall kidney health, as shown by normalization of urine ACR, serum albumin, blood urea nitrogen (BUN), and creatinine to WT levels (Figure 9, A–D), and protected kidney function (Supplemental Table 4). Treating SPL^R222Q^ mice with the selective SphK1 inhibitor PF543 under the same conditions gave similar results, indicating the pool of S1P responsible for inducing kidney injury was largely, if not completely, generated by SphK1 (Figure 9, A–D, and Supplemental Table 4). Importantly, genes upregulated in the kidneys of SPL^R222Q^ mice on NA-B6 chow were normalized by preventing S1P accumulation using SKI-II and to a lesser extent PF543 (Supplemental Figure 6). Pathological changes evident in the cortex of SPL R222Q mice on NA-B6 diet were also attenuated when these mice were treated with either of the SK inhibitors (Figure 9E). These findings indicate that the transcriptional reprogramming of cytokines and ECM proteins associated with SPLIS nephrosis is mediated by S1P accumulation.

To determine whether S1P receptor signaling mediates SPLIS nephrosis, SPL^R222Q^ mice were given NA-B6 chow for 2 months and then treated for 2 weeks with FTY720, which functionally antagonizes S1P1, S1P3, S1P4, and S1P5, or with JTE-013, which antagonizes S1P2. The findings of worsening lymphopenia (a sign of S1P1 antagonism; Supplemental Table 3) and reduced activation of S1P receptor downstream effectors ERK, JNK, STAT3, and AKT in the kidney tissues of WT and SPL^R222Q^ mice treated with FTY720 (Supplemental Figure 7) confirmed the receptor-antagonizing activity of these agents. However, blocking S1P signaling with FTY720 or JTE-013 did not prevent the development of SPLIS nephrosis in SPL^R222Q^ mice and may even have exacerbated it (Figure 9, A–E, and Supplemental Table 4). Furthermore, FTY720 exacerbated Ccl17 upregulation; did not alter Ccl12, IL-6, and Tnfsf15 upregulation; and only attenuated TNF-α and Grem2 upregulation (Supplemental Figure 6). To address whether FTY720 might have effects on the kidney independent of SPL status, we treated WT mice on both diets with FTY720. Urine ACR was higher in FTY720-treated WT mice fed either type of diet (Supplemental Table 4). Taken together, these results support the conclusion that a SphK1/S1P pathway drives kidney injury in SPLIS, whereas the role of S1P receptors remains unclear.

### SPLIS nephrosis involves the RhoA/ROCK signaling pathway.

We recently reported that when kidney cells fail to contain the metabolic consequences of SPL insufficiency, massive urinary S1P excretion and renal S1P accumulation occur, causing RhoA/ROCK-mediated cytoskeletal reorganization and loss of cell function (51). Furthermore, blocking this pathway with fasudil reduced proteinuria in *Sgpl1*-KO mice. The RhoA/ROCK pathway was upregulated in SPL^R222Q^ mouse kidneys on NA-B6 chow (Supplemental Figure 5). To assess the relative contributions of RhoA/ROCK activation to the kidney disease in SPL^R222Q^ mice, we treated them with fasudil. As shown in Figure 9 and Supplemental Table 4, fasudil protects SPL^R222Q^ mice from developing proteinuria when on low B6 chow, implicating the same mechanism is at play in both SPLIS models.

## Discussion

SPLIS is a recently recognized inborn error of metabolism predicted to afflict thousands of individuals worldwide. SPLIS-associated nephrosis usually progresses rapidly to renal failure and is, directly or indirectly, the primary cause of childhood deaths in SPLIS (4). While kidney transplantation can be therapeutic in SPLIS patients with end-stage kidney disease, the procedure is invasive and requires long-term immunosuppression, and many SPLIS children are not suitable surgical candidates (4). Furthermore, kidney transplantation may not address the extrarenal manifestations of SPLIS. Alternative therapies are direly needed, particularly targeted treatments that address the root cause of SPLIS and have the potential to prevent or delay kidney failure and, ideally, other manifestations of this heterogeneous condition.

Vitamin B6 supplementation has been used successfully to treat deficiencies of PLP-dependent enzymes. PLP cannot be imported readily by cells, whereas cells can import pyridoxine and convert it intracellularly to PLP. Pyridoxine therapy increases intracellular PLP levels, thereby overcoming the reduced cofactor-binding affinity and/or improving the protein stability of some pathogenic PLP-dependent enzyme variants. Pyridoxine is given orally and exhibits an acceptable safety profile in infants and pregnant women (52). Despite these attributes, pyridoxine’s therapeutic activity is limited to a subset of variants in each inborn error of metabolism. This necessitates testing variants (or patients) individually. Furthermore, pyridoxine treatment at doses greater than 500 mg/d may result in a sensory peripheral neuropathy (53). Demonstration that pyridoxine supplementation affords metabolic correction and disease-modifying activity in preclinical SPLIS models would provide the rationale for long-term pyridoxine treatment and monitoring.

In this study, we employed 3 complementary approaches to establish proof of concept for the efficacy of cofactor supplementation in SPLIS. We focused on the SPL R222Q variant, as it is the most common SPLIS-associated pathogenic variant and its low expression, activity, and predicted cofactor binding affinity make it a candidate for both prosthetic and chaperone functions of PLP (4, 20). In addition, we had observed clinical improvement in response to pyridoxine in a patient with SPLIS homozygous for the SPL R222Q variant. We also showed pyridoxine restored sphingolipid metabolism in skin fibroblasts derived from patients with the R222Q variant (20). Here, we show the peripheral neuropathy of a second patient with R222Q-variant SPLIS abated after pyridoxine administration, objectively measured by NCS. This occurred concomitant with a reduction in plasma S1P levels and was consistent with increased SPL activity after pyridoxine treatment in patient-derived skin fibroblasts. Furthermore, purified recombinant human SPL R222Q exhibited a dose-dependent increase in catalytic activity in response to PLP. In contrast, PLP exerted no effect on an SPL variant in which tryptophan, rather than glutamine, is substituted for R222 (54). Additional PLP also had no effect on WT SPL, which binds PLP optimally during polypeptide synthesis and assumes a thermodynamically favorable (native) protein conformation. Thus, the SPL holoenzyme would not be expected to benefit catalytically or be further stabilized by cofactor supplementation. Thus, our in vitro findings demonstrate the R222Q variant requires a higher concentration of PLP for its catalytic activity than WT. Molecular modeling of the SPL protein showed that substitution of glutamine for arginine would cause misalignment of helix 243–252, a key determinant of PLP binding, with the consequence of reduced cofactor binding affinity. We used Boltzmann’s equation to calculate that a 100- to 200-fold increase in PLP (the range of pyridoxine supplementation dosing in humans) would likely rescue the R222Q variant from the impact of the substitution on the energetic stability of the variant. While we consider this a plausible mechanism for pyridoxine supplementation’s efficacy in this situation, it remains to be tested experimentally. Although pyridoxine only raises SPL R222Q-variant enzyme activity to about 25% of healthy control levels, this amount of activity has been shown to prevent the consequences of SPL insufficiency in animal models (21). This result establishes the biochemical basis for pyridoxine supplementation in SPL R222Q-variant SPLIS.

To further explore the impact of pyridoxine administration on tissue SPL activity, sphingolipid metabolism, organ function, and pathology in SPLIS, we used gene editing to generate the SPL^R222Q^ mouse, a customized knockin animal model of SPL R222Q-variant SPLIS. Despite the low SPL activity observed in tissues of SPL^R222Q^ mice fed Hi-B6 chow, which ranged from 25%–50% of WT levels, they did not accumulate S1P, indicating this level of activity is sufficient for proper sphingolipid metabolism. Similarly, no SPLIS-associated phenotypes were detected under these conditions. However, upon withdrawal of pyridoxine supplementation, SPL^R222Q^ (but not WT) mice showed metabolic, neurological, renal, and hematological deterioration. Thus, the SPL^R222Q^ mouse represents a unique inducible SPLIS model with advantages over the *Sgpl1*-KO model. Because it can reach adulthood, conducting complex procedures and assessing the efficacy of therapeutic agents that act by modifying the activity or stability of the variant are possible in SPL^R222Q^ mice. Our results simultaneously confirmed that the SPL R222Q variant is pyridoxine responsive and that progression of kidney disease should be preventable in SPLIS patients harboring pyridoxine-responsive pathogenic variants of SPL.

Histological and molecular methods combined with transcriptional profiling were used to characterize the impact of SPL insufficiency on murine kidneys. Our kidney transcriptome results demonstrate that inactivation of the SPL R222Q variant led rapidly to kidney injury, as evidenced by upregulation of *Kim1/Havcr1*, a well-established marker of kidney injury which is also reported to influence lipid uptake in proximal tubule cells (38). Loss of SPL function also led to activation of the innate immune system, including effects on TNF-α, IL-17, TGF-β/SMAD, other chemokines and cytokines, macrophage polarization, and complement activation. A prominent signature of ECM remodeling presaged the later development of kidney fibrosis. Our IB results showed that SPL insufficiency led to activation of STAT3, AKT, MAPK/ERK, PKCδ, and TGF-β, stress pathways that are known downstream effectors of both cytokines and S1P/SPL. Pathological results confirmed the essential role of SPL in kidney homeostasis, as demonstrated by podocyte injury, podocyte loss, glomerular injury, protein casts, and kidney fibrosis when SPL was inactivated.

S1P is generated through phosphorylation of the precursor sphingosine by either SphK1 or SphK2. SphK1 is largely cytosolic and can translocate to the plasma membrane upon stimulation, producing a pool of S1P that can be exported to engage S1P receptors in an autocrine/paracrine manner (55). SphK2 is enriched in intracellular compartments (nucleus, endoplasmic reticulum, and mitochondria) and is frequently linked to regulation of intracellular S1P pools that can influence transcriptional programs and stress responses (55). By inhibiting S1P production using either a nonspecific or selective inhibitor of SphK1, kidney protection was afforded to SPL^R222Q^ mice under conditions that uniformly lead to SPLIS nephropathy. Importantly, the transcriptional changes associated with SPLIS nephrosis that we observed in the SPL^R222Q^ mouse kidneys were completely prevented by inhibiting S1P production, which directly implicates S1P accumulation as the causal factor in kidney injury. We observed a modest increase in efficacy of the nonspecific inhibitor over the selective SphK1 inhibitor PF543 based on ACR normalization. There are several possible explanations for this. First, the nonselective inhibitor SKI-II also impacts the activities of SphK2 and dihydroceramide desaturase (56). These enzymes could potentially contribute to SPLIS nephropathy in a minor way. A more likely explanation is the fact that SKI-II induces proteolytic degradation of SphK1, which could have a more lasting and profound effect on SphK1 inhibition beyond its catalytic effects (57). Regardless of these possibilities, the major effect is clearly mediated by an SphK1-generated pool of S1P. S1P mediates many of its biological effects through activation of its receptors (58). However, S1P receptor antagonism did not prevent SPLIS nephropathy, and FTY720 only blocked the upregulation of 2 cytokines, TNF-α and Grem2. This finding could be explained by S1P’s receptor-independent functions, such as calcium signaling, epigenetic regulation of gene expression through its effect on histone deacetylases, DNA repair and telomere maintenance, and mitochondrial function (59–61). However, this mechanism cannot be concluded with certainty, as FTY720 and JTE-013 both have some negative effects on kidney function, as our control experiments demonstrated. Finally, in addition to the role of S1P, we demonstrate that RhoA/ROCK signaling, a key regulator of cytoskeletal dynamics, is involved in mediating the development of nephrotic syndrome in the SPL^R222Q^ mouse model, as the ROCK inhibitor fasudil was protective, similar to its effect in a global *Sgpl1*-KO mouse model, as we recently reported (51).

Some caveats to our study should be noted. We did not carry out the study for an extended period. Thus, we cannot rule out the possibility that kidney disease or other phenotypes might eventually develop in SPL^R222Q^ mice under Hi-B6 conditions. Some SPLIS phenotypes, such as glucocorticoid deficiency, hypercholesterolemia, and reproductive health, were not assessed in our model. These will be evaluated in future studies. Clinical studies testing the efficacy of pyridoxine will be needed to validate our preclinical findings. Importantly, although SPL R222Q represents the most prevalent pathogenic SPL variant in SPLIS, many other variants have been identified, including many missense variants with the potential to be activated by pyridoxine supplementation (4). Experimental testing of these variants is necessary before extrapolating our findings.

In conclusion, our findings using a personalized murine knockin mouse model of SPL^R222Q^ SPLIS demonstrate that pyridoxine supplementation improves tissue SPL activity, reduces tissue and plasma S1P levels to near normal levels, and prevents nephrosis, glomerulosclerosis, and other SPLIS phenotypes.

## Methods

### Sex as a biological variable.

In this study, plasma S1P levels in 1 female patient with the rare disease SPLIS were compared with the S1P levels in healthy individuals of both sexes. SPLIS incidence is equal in both sexes (4). In animal studies, we used male and female mice with roughly equal distribution in all treatment groups. Based on years of observation, we have no evidence that sex influences the development or severity of kidney, neurological, or immunological phenotypes in SPLIS or SPLIS mouse models. Thus, we believe our findings are equally relevant to both sexes.

### WES.

Blood samples, clinical data, and fibroblasts were obtained from the proband after obtaining informed consent. DNA for WES and plasma for sphingolipid analysis before and after pyridoxine treatment were obtained from blood. The quality of the DNA was evaluated by 1% agarose gel electrophoresis. The DNA libraries for sequencing were constructed according to the manufacturer’s protocol. Subsequently, we conducted the sequencing on an Illumina HiSeq 2000 platform. Sequencing reads were aligned to the human genome (GRCh37/hg19). SAM files from each sample were then converted to BAM format, sorted, and merged using SAMtools (http://samtools.sourceforge.net/). PCR duplicates were removed from the files using Picard (https://broadinstitute.github.io/picard/). Further processing was carried out utilizing the Genome Analysis Toolkit (GATK) from the Broad Institute (http://www.broadinstitute.org/gatk/). All BAM files underwent local realignment with an insertion/deletion realigner. Single-nucleotide variants and insertions/deletions within the captured coding exonic regions were identified using GATK’s unified genotype. The detected variants were filtered according to the criteria that we previously reported (1). Only the *SGPL1* variant (c.887C>A, R222Q) was segregated in the proband, following a recessive inheritance pattern. The conservation of mutations was assessed using ClustalW.

### Isolation and propagation of skin fibroblasts.

Primary fibroblasts were isolated from the SPLIS subject as described (62) and transduced with *hTERT* using lentivirus vector pLV-hTERT-IRES-hygro (Addgene plasmid 85140). Control (CCD32) skin fibroblasts were from the University of California, Berkeley, Cell Culture Facility. Hygromycin (100 μg/mL) was introduced for selection 2 days after transduction. In some cases, retroviral vector pBABE-puro-hTERT (Addgene plasmid 1771) and puromycin selection (at 1–2 μg/mL) were employed to establish immortalized fibroblasts. Following identification of stable cells, immortalized fibroblast lines were expanded and subcultured for subsequent experiments.

### SPL activity and sphingolipid quantification.

Recombinant SPL activity was measured by reacting 10 μM S1P (Avanti Polar Lipids) in PBS with 50 nM purified enzyme and varying concentrations of PLP. Reactions were quenched by mixing 1:1 with 2× 1,2-diacetylbenzene (DAB) buffer (2×: 500 mM sodium bicarbonate pH 10.0, 2% SDS, 2.5 mM β-mercaptoethanol, and 2.5 mM DAB [Sigma-Aldrich]), and DAB fluorescence upon reaction with the SPL product ethanolamine phosphate was measured at excitation/emission wavelengths of 355 nm/455 nm after 1 hour. SPL activity was measured in human skin fibroblasts and tissues of SPL^R222Q^ mice by monitoring formation of the (2*E*)-hexadecenal product using liquid chromatography–tandem mass spectrometry (63). For quantification of S1P in murine tissues and plasma, lipid extraction was performed, followed by separation and identification using tandem mass spectrometry on an Agilent 6495C triple quadrupole instrument as described (64).

### NCS.

NCSs were conducted utilizing a Dantec Keypoint Portable Nerve Conduction/Electromyography machine from Dantec Dynamics. The findings were analyzed and reported by a neurophysiologist, and conduction defects were categorized in accordance with the neurophysiological classification of carpal tunnel syndrome.

### Construction of R222Q and R222W variants of the SGPL1 gene.

Recombinant WT *SGPL1* sequence was purchased as an *E*. *coli* codon-optimized gBlock from Integrated DNA Technologies. End primers were designed to remove the first 92 N-terminal residues and insert a C-terminal 6xHis tag with a GSG linker (Supplemental Table 5). The insert was digested with NdeI and XhoI and ligated into the respective sites in the vector pET22b. R222 mutations (Q and W) were introduced using overlap extension PCR, and all constructs were confirmed by sequencing with T7 primers.

### Protein expression and purification.

WT, R222Q, and R222W SPL variants were introduced into *E*. *coli* BL21 (DE3) containing helper plasmid pG-KJE8 (Takara) and grown at 37°C before inducing with 0.1 mM IPTG and 0.2% arabinose and expressing overnight at 25°C. Expression cultures were pelleted and stored at –20°C until the day of purification. Purification was performed as described previously (65). Briefly, cells were lysed in buffer containing excess PLP (20 mM Tris, 300 mM NaCl, 10 mM imidazole, 15% sucrose, 1 mM PLP, and 0.125 mM PMSF, pH to 8.0 with HCl) and loaded on a Ni-NTA affinity column. Enzyme was eluted under low-salt (10 mM NaCl) conditions and subsequently cleaned up by cation exchange chromatography (UNOsphereS, Bio-Rad). PLP was included in the initial step of purification to ensure formation of the holoenzyme but was excluded from later steps to determine the effect of supplementation in vitro.

### Animals.

All animal studies were conducted under approved IACUC protocols at the NIH and at UCSF. To generate SPL^R222Q^ mice, we introduced the R222Q mutation into exon 7 of the mouse Sgpl1 gene using CRISPR/Cas9 editing technology as described previously (66). We designed an sgRNA containing a 20 bp target sequence, 5′-GATGGCCTGCAAAGCTTACC-3′ (Synthego), on mouse exon 7. To introduce the mutant sequence, we designed a single-stranded 199-base oligodeoxynucleotide (SPLIS ssOligo) containing the *SGPL1*c.665G>A SPLIS variant, flanked by 92 bases on the 5′ side and 106 bases on the 3′ side: 5′-AGGCTGTACCTCCTGATACCCATCCTTAACTTACTCTGGTTTCTTTTCTTCACATAGGTGACTTCTGGGGGAACGGAAAGCATCCTGATGGCATGCAAAGCTTACCAGGACTTGGCGTTAGAGAAGGGGATCAAAACTCCAGAAATGTATGGATGTGTGTGTTTGTTTCCCTTCTGATATTGTCTATTTGTGGCAGCAC-3′ (Integrated DNA Technologies). The SPLIS ssOligo also contained a silent change to create a SphI restriction enzyme site for screening. The C57BL/6J strain was used as an embryo donor. Fertilized oocytes were injected with Cas9 protein (TrueCut Cas9 protein V2, Thermo Fisher Scientific), sgRNA, and SPLIS ssOligo and transferred to the oviducts of pseudopregnant female mice. Founder mouse was confirmed by Sanger sequencing. Mice were genotyped by PCR of tail-snip DNA at weaning using the flanking primer 1, 5′-AAGACTAGAATTGTAGG-3′, and primer 2, 5′-CCTCCTACAATTCTAGTCTT-3′, under the following PCR conditions: denaturation at 95°C for 30 seconds, amplification at 55°C for 1 minute, and extension at 72°C for 1 minute (40 cycles). The resulting PCR product (443 bp) was digested with SphI to identify potential founders carrying the R222Q change. WT allele yielded a 443 bp, undigested fragment, and the SPLIS ssOligo-modified allele yielded 275 and 167 bp bands. Mice were fed chow with Hi-B6 (18 mg/kg) content or NA-B6 (AIN-93 chow with ~2 ppm pyridoxine, LabDiet). Pyridoxine content was determined by the Nestle Purina Analytical Lab.

### IB.

Kidneys from SPL^R222Q^ and WT mice on NA-B6 and Hi B6 chow were homogenized and cleared by centrifugation. The following antibodies for IB analysis were purchased from Cell Signaling Technology: anti–phosphorylated Stat3 (catalog 9145), anti–total STAT3 (catalog 9131), p-ERK1 (catalog 4370), t-ERK1 (catalog 4696), p-PKCδ (catalog 9376), t-PKCδ (catalog 2058), p-AKT (catalog 4060), t-AKT (catalog 9272), p-SMAD3 (catalog 9520), and t-SMAD3 (catalog 9513). Synaptopodin (sc-515842) and nephrin (sc-376522) antibodies were purchased from Santa Cruz Biotechnology. Antibodies against murine SPL were produced in-house as described previously (22), and antibodies against β-actin (SAB5600204) were purchased from Sigma-Aldrich. Antibodies against human SPL (AF5535) were from R&D Systems. HRP-conjugated secondary antibodies were as follows [115-035-003, Goat Anti-Mouse IgG (H + L); 111-035-144, Goat Anti-Rabbit IgG (H + L), Jackson ImmunoResearch; sc-2020, donkey anti-goat IgG-HRP, Santa Cruz Biotechnology]. Signals were detected using the SuperSignal West Pico kit (Thermo Fisher Scientific). Radiographic bands were quantified using ImageJ (NIH).

### Hematological and urine ACR analysis.

Serum albumin and whole-blood red blood indices were measured at University of California, Davis, Comparative Pathology Laboratory. Briefly, 100 μL of diluted blood samples were analyzed using the XT-2000iV veterinary hematology analyzer (Sysmex) to obtain a full standard hematology profile, and a COBAS INTEGRA 400 plus instrument (Roche Diagnostics) was used to assess serum albumin levels.

### PAS staining and IHC.

Kidneys were fixed in 4% paraformaldehyde, paraffin embedded, and sectioned to 5 μm thick. PAS staining and IHC were performed as described previously (23, 67). Qualitative evaluation was performed by a nephropathologist to identify segmentally sclerosed glomeruli and glomeruli displaying mesangial hypercellularity and interstitial fibrosis. For IHC, p-STAT3 (Tyr705) (D3A7) XP rabbit monoclonal antibody (Cell Signaling Technology, catalog 9145) was used. All images were captured using a Nikon Eclipse 80i microscope equipped with a digital camera (Nikon DS-Ri1).

### STED super-resolution fluorescence microscopy.

STED was performed at the University of Southern California (USC) Stem Cell Optical Imaging Facility on paraffin-embedded fixed kidney cortex sections. For antigen retrieval, heat-induced epitope retrieval with sodium citrate buffer (pH 6.0) was applied. To reduce nonspecific binding, sections were blocked with goat serum (1:20, Sigma-Aldrich, catalog G9023). For tissue permeabilization, samples were incubated with 0.1% Triton X-100 for 10 minutes. Other samples were incubated with primary anti-nephrin (1:50, Progen, catalog GP-N2) and STED-compatible secondary Alexa Fluor 488 (1:500, Invitrogen, catalog A-11073) antibodies, mounted in Prolong Diamond media (Invitrogen, catalog P36961), and covered with number 1.5 glass coverslips. STED xyz images were acquired using a Stellaris 8 STED microscope (Leica Microsystems) equipped with an HC PL APO ×100/1.4 OIL STEDWHITE objective. Pixel sizes of 20–25 nm were used for STED nanoscopy with line accumulation set to 4. The pinhole was set to 1.0 AU. Anti-nephrin–Alexa Fluor 488 was excited at 492 nm, and STED was performed using a pulsed depletion laser at 592 nm. For both colors, acquisition time gating was set to 1–10 ns.

### Transmission electron microscopy.

Electron microscopy sample handling and detection were performed by the Core Center of Excellence in Nano Imaging at USC. The foot process width and the number of foot processes per micrometer of glomerular basement membrane were calculated using a curvimeter (Sakura Co.). Five glomeruli were randomly selected from each mouse; 10 electron micrographs were taken and analyzed for each glomerulus.

### Grip strength testing.

Each mouse underwent 3 sets of grip strength tests using a Chatillon Ametek digital force gauge (model DFIS-10) as described previously (23).

### RNA extraction and RNA-seq.

Total RNA was extracted from kidney tissues using TRIzol reagent (Thermo Fisher Scientific) according to the manufacturer’s protocol. RNA integrity was verified using an Agilent Technologies 2100 Bioanalyzer. Some RNA was used for qPCR described below. For RNA-seq studies, ribosomal RNA was removed, followed by fragmentation using divalent cation buffers at elevated temperatures. The sequencing library was prepared following Illumina’s TruSeq stranded total RNA sample preparation protocol. Quality control analysis and quantification of the sequencing library were performed using Agilent Technologies 2100 Bioanalyzer High Sensitivity DNA Chip. Paired-end sequencing was performed on Illumina’s NovaSeq 6000 sequencing system.

### Transcript assembly and differential expression analysis of transcripts.

RNA-seq was performed by LC Sciences. Cutadapt (https://cutadapt.readthedocs.io/en/stable/) and customized Perl scripts were used to remove the reads that contained adaptor contamination, low-quality bases, and undetermined bases. The sequence quality was verified using FastQC (https://www.bioinformatics.babraham.ac.uk/projects/fastqc/). We used HISAT2 (68) to map reads to the genome of *Mus musculus* (version 101). The mapped reads of each sample were assembled using StringTie (69). Then, all transcriptomes from 12 samples were merged to reconstruct a comprehensive transcriptome using Perl scripts and gffcompare (commit ID LCS9956; https://github.com/gpertea/gffcompare/).After the final transcriptome was generated, StringTie (69) and ballgown (http://www.bioconductor.org/packages/release/bioc/html/ballgown.html) were used to estimate the expression levels of all transcripts. StringTie was used to calculate the expression level of mRNAs and long noncoding RNAs (lncRNAs) by calculating fragments per kilobase of transcript per million mapped fragments (FPKM). mRNA and lncRNA differential expression analysis was performed using DESeq2 software (70) between 2 different groups (and by edgeR between 2samples) (71). The genes/mRNAs/lncRNAs with FDR below 0.05 and absolute fold change ≥ 2 were considered differentially expressed.

### GSEA.

We performed GSEA using GSEA software (v4.1.0) (72) and MSigDB to identify whether a set of genes in specific GO terms or KEGG pathways showed significant differences between the 2 groups. Enrichment scores and *P* values were calculated using the default parameters. GO terms and KEGG pathways meeting this condition with normalized enrichment score > 1, *P* value < 0.05, and FDR *q* value < 0.25 were considered different between the 2 groups. GO terms were calculated using the hypergeometric equation. GO terms with *P* value < 0.05 were defined as significant.

### qPCR.

cDNA synthesis was performed by using SuperScript III reverse transcriptase (Thermo Fisher Scientific). qPCR was performed using PowerUp SYBR Green Master Mix in the QuantStudio 6 and 7 Flex Real-Time PCR System (Life Technologies, Thermo Fisher Scientific). Cq values of samples were normalized to the corresponding Cq values of *Actb*. The comparative Cq method was used to determine the quantification of the fold change in gene expression. Primers are listed in Supplemental Table 5.

### Antagonists and inhibitors.

SPL^R222Q^ mice were given NA-B6 chow for 2 months. Additional groups of WT mice given Hi-B6 and NA-B6 and SPL^R222Q^ mice given Hi-B6 for 2 months were used as controls. SPL^R222Q^ mice on NA-B6 were then treated for 2 weeks with either the S1P_1,3,4,5_ receptor antagonist FTY720 (Cayman Chemical) at 0.5 mg/kg, the specific SphK1 inhibitor, PF543 (Selleck Chemicals) at 1 mg/kg, the broad-spectrum SphK1/2 and dihydroceramide desaturase inhibitor SKI-II at 50 mg/kg (Cayman Chemical), the S1P2 receptor antagonist JTE-013 at 10 mg/kg (Fisher Scientific), the ROCK inhibitor fasudil at 2 mg/kg (Sigma-Aldrich), or vehicle (DMSO in normal saline) given i.p. every other day. WT mice on both diets were also treated with FTY720 to evaluate the impact on kidney function of this broad-spectrum S1P receptor antagonist. All agents were dissolved in DMSO stock solution. After the last dose, urine was collected after 24 hours. Two days after the last dose, the mice were euthanized with terminal phlebotomy for serum albumin, creatinine, and BUN. Both sexes were used for all treatment groups.

### Statistics.

Data analysis was conducted with GraphPad Prism 5 (GraphPad Software). The individual statistical methods are mentioned in each figure. A *P* value < 0.05 was considered significant.

### Study approval.

Written informed consent was received prior to human subject participation in the study. Informed consent was obtained in accordance with a protocol approved by the UCSF IRB. Healthy pediatric and young adult patients undergoing elective surgeries for benign conditions were recruited from the outpatient surgery center at the UCSF Benioff Children’s Hospital, Oakland, California, USA. Any patients with malignant, infectious, metabolic, genetic, hemolytic, autoimmune, or endocrine disorders were excluded. Plasma samples with visible hemolysis were also excluded. All animal protocols were approved by the Institutional Animal Care and Use Committee at UCSF and USC.

### Data availability.

RNA-seq data are available in the supplemental material and have been deposited in the Gene Expression Omnibus repository under accession number GSE345689. Raw data are available in the Supporting Data Values file.

## Manuscript information

### Author contributions.

JDS designed the research studies, analyzed the data, and, together with RK, wrote the manuscript. RK, MLA, EK, JYL, MRS, AI, VB, GG, JPP, RAK, GT, BO, RLP, AC, ES, JBH, YTC, and RZ conducted experiments, acquired and analyzed data, provided reagents, and edited the manuscript. All authors approved the final version of the manuscript.

### AI disclosure.

The authors declare that no AI tools were used in the original research for or writing of this article.

### Conflict of interest.

JDS is an inventor on the patents “Adeno-Associated Viral (Aav)-Mediated Sgpl1 Gene Therapy for Treatment of Sphingosine-1-Phosphate Lyase Insufficiency Syndrome (Splis)” and “Sphingolipid Biomarkers for Sphingosine Phosphate Lyase Insufficiency Syndrome and Related Conditions.” ES, MRS, and RAK are inventors of intellectual property related to S1P lyase.

### Funding.

This study was supported by the following:

- Intramural Research Program of the National Institute of Diabetes and Digestive and Kidney Diseases within the NIH.
- NIH Public Health Service grants R01HD113778, R21OD037868, and R21TR004262 to JDS and R01DK064324 and R01DK123564 to JPP.
- California Institute of Regenerative Medicine grant DISC2-13072 to JDS.
- NIH U2C/TL1 LAUNCH grant postdoctoral fellowship (5TL1DK139565-03) to RK.

This work is the result of NIH funding, in whole or in part, and is subject to the NIH Public Access Policy. Through acceptance of this federal funding, the NIH has been given a right to make the work publicly available in PubMed Central.

## Supplementary Material

Supplemental data

Unedited blot and gel images

Supporting data values

## Figures and Tables

**Figure 1:**
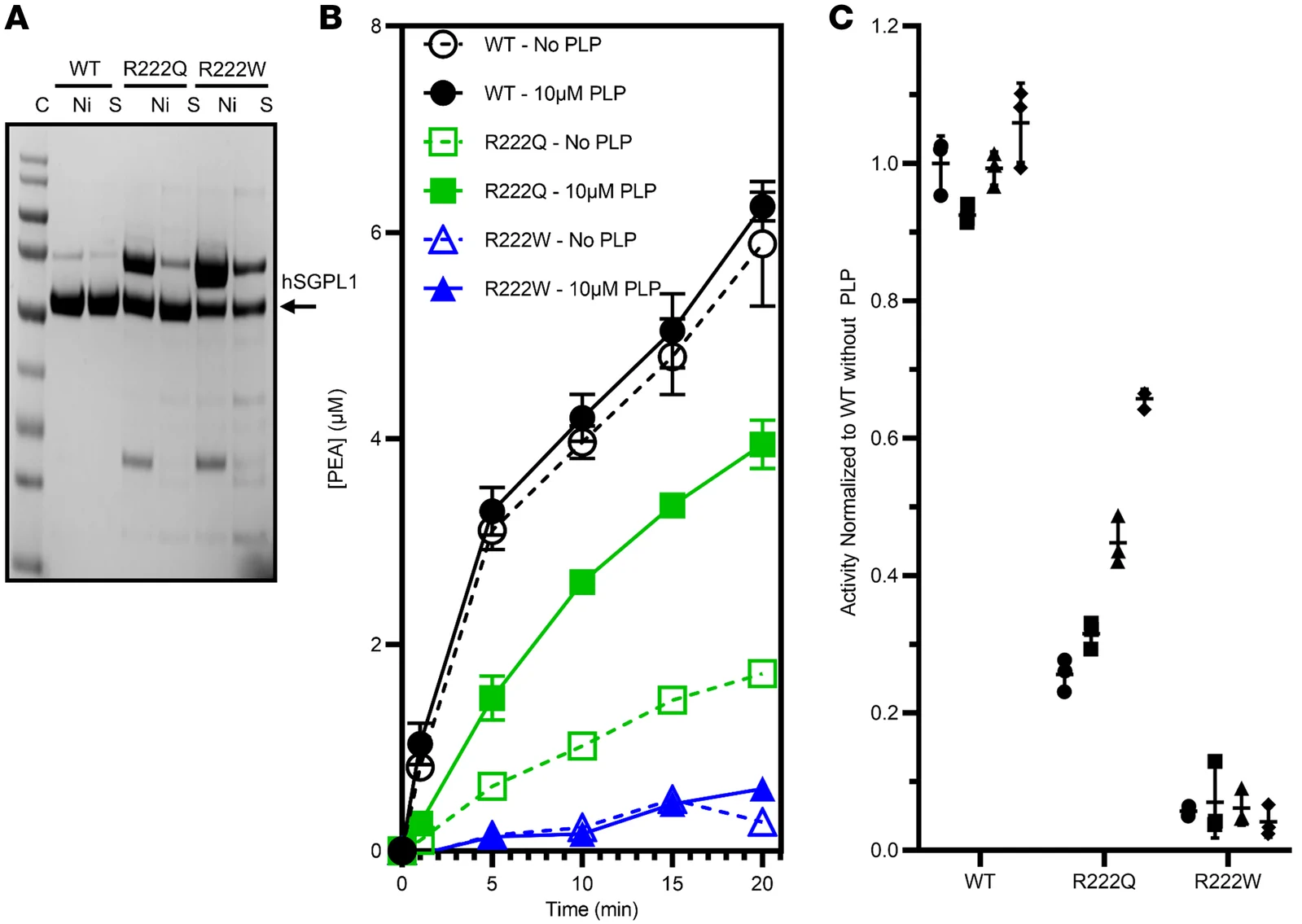
SPL protein purification, stability, and enzymatic activity. (**A**) Coomassie-stained SDS-PAGE gel of purified human S1P lyase showing elutions from affinity (Ni) and ion-exchange (S) columns. C, results normalized to control. (**B**) Time-course reaction of 50 nM purified S1P lyase with 10 μM S1P with indicated concentrations of PLP. Data represent the SD of triplicate measurements. *P* values: WT 0 μM PLP versus 10 μM = NS, R222Q 0 μM PLP versus 10 μM = 0.00353, and R222W 0 μM PLP versus 10 μM = NS. PEA, phosphoethanolamine. (**C**) Activity of enzymes at 5 minutes normalized to the WT enzyme without PLP. Data represent mean ± SD. Enzyme activity with 10 μM PLP: WT versus R222Q < 0.0001, WT versus R222W < 0.0001, and R222Q versus R222W < 0.0001. A 2-tailed parametric *t* test was performed.

**Figure 2:**
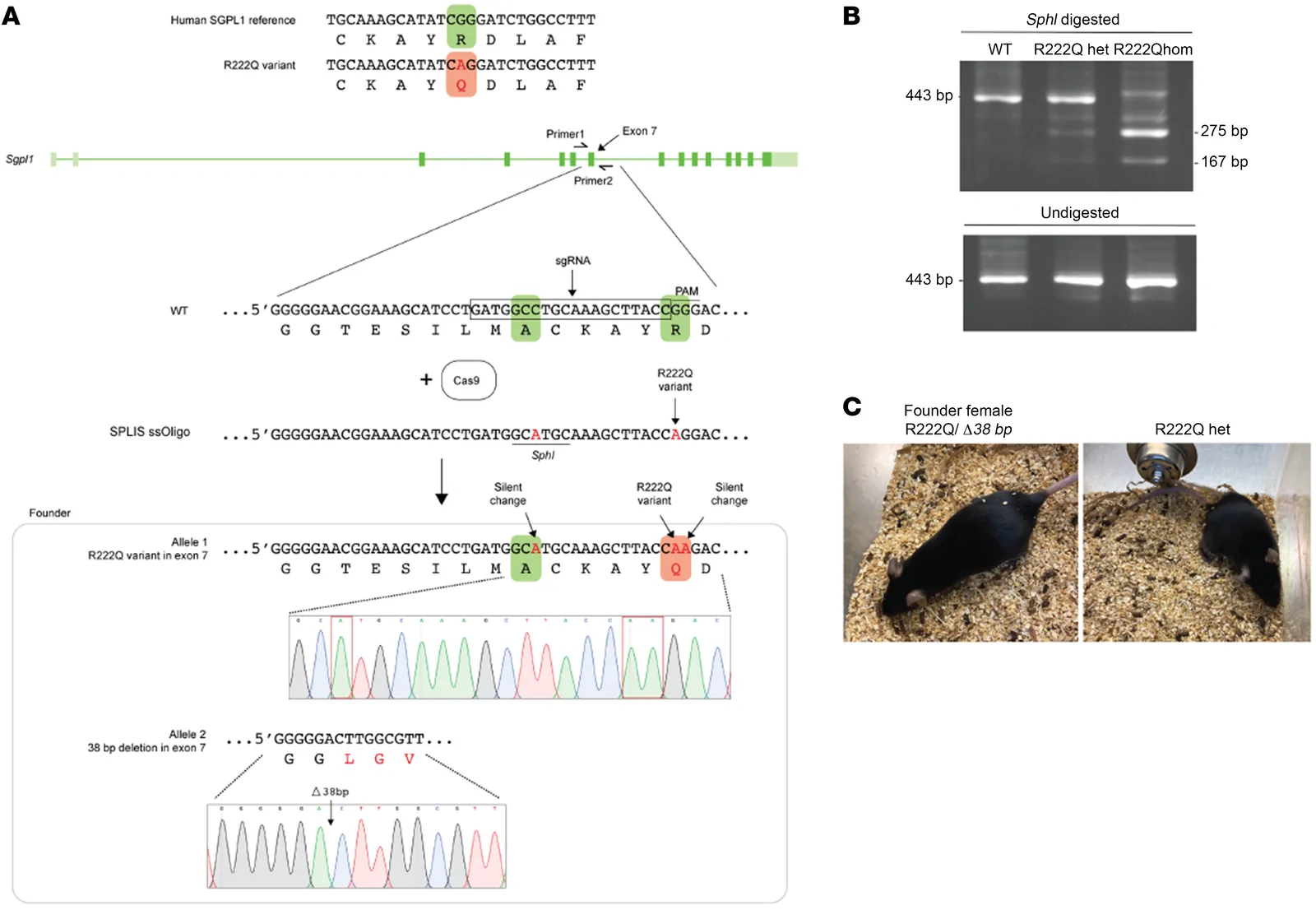
Generation of SPLIS R222Q mutant mice. (**A**) An sgRNA containing a 20 bp target sequence, 5′ GATGGCCTGCAAAGCTTACC 3′ (Synthego) of exon 7. To introduce the mutant sequence, we designed a single-stranded 199-base oligodeoxynucleotide (SPLIS ssOligo) containing the G-to-A SPLIS mutation and a silent point mutation to create a SphI restriction enzyme site for screening: 5′-AGGCTGTACCTCCTGATACCCATCCTTAACTTACTCTGGTTTCTTTTCTTCACATAGGTGACTTCTGGGGGAACGGAAAGCATCCTGATGGCATGCAAAGCTTACCAGGACTTGGCGTTAGAGAAGGGGATCAAAACTCCAGAAATGTATGGATGTGTGTGTTTGTTTCCCTTCTGATATTGTCTATTTGTGGCAGCAC-3′ (Ultramer DNA, Integrated DNA Technologies). C57BL/6J strain was used as an embryo donor. Fertilized oocytes were injected with Cas9 protein (TrueCut Cas9 protein V2, Thermo Fisher Scientific), the sgRNA, and the SPLIS ssOligo and transferred to the oviducts of pseudopregnant female mice. (**B**) Mice were genotyped and the resulting PCR product (443 bp) digested with SphI to detect the mutant sequence. After digestion, the WT allele yielded a 443 bp fragment; the SPLIS-modified allele yielded 275 and 167 bp bands. We detected 1 founder whose PCR fragment was digested by SphI (out of 99 offspring screened). Sequences of the undigested PCR fragments were determined after TOPO TA cloning (Thermo Fisher Scientific) by Sanger sequencing. The founder female carried 1 allele containing the R222Q mutation and SphI restriction site and 1 allele with a 38 bp deletion in exon 7, resulting in a frameshift and premature stop codon (**A** and **B**). (**C**) When mated with a WT male, the founder produced 4 pups, 2 *Sgpl1* R222Q/WT and 2 *Sgpl1* del/WT, segregating the 2 alleles.

**Figure 3:**
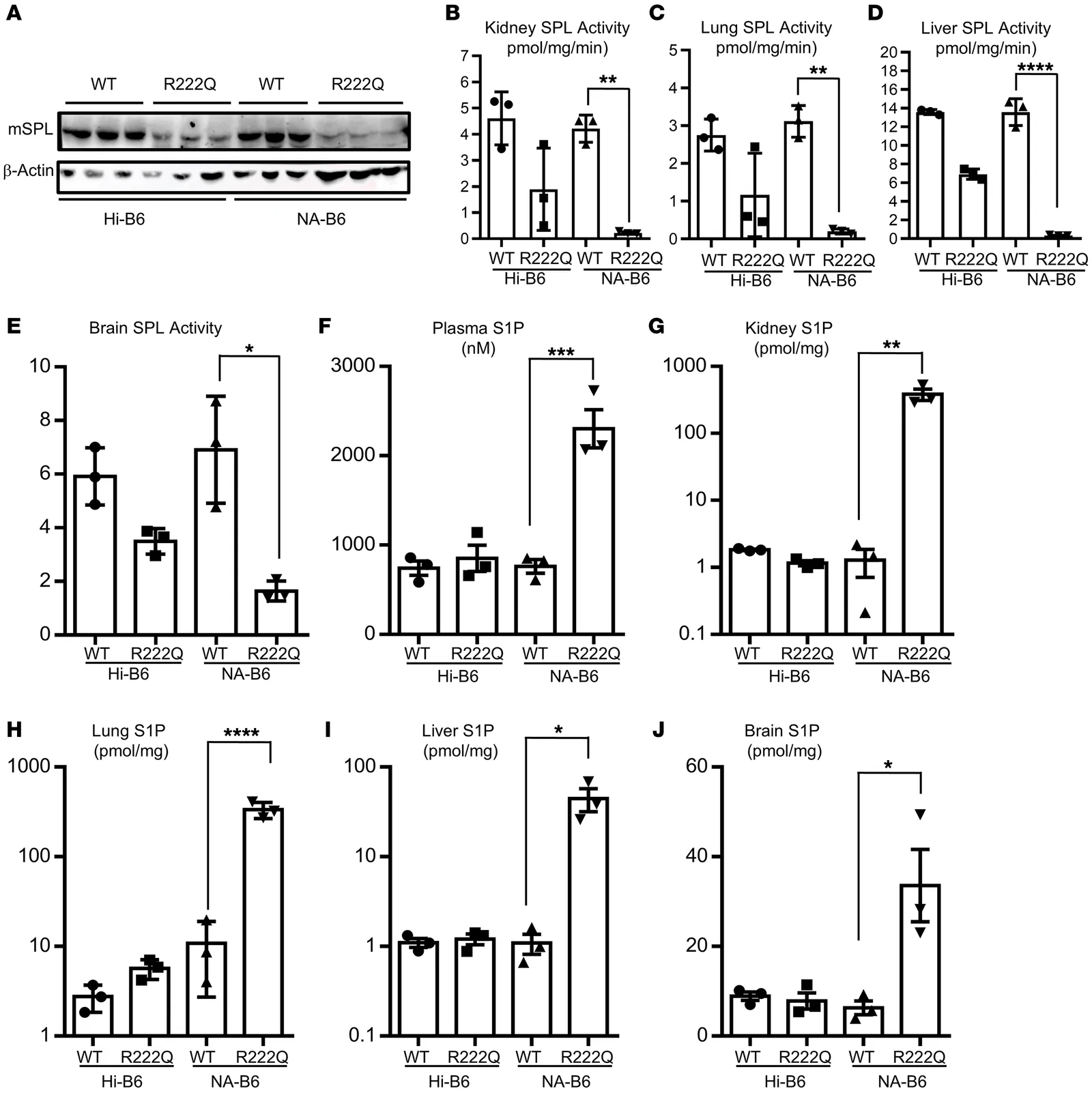
SPL^R222Q^ mice on NA-B6 manifested severe SPL insufficiency and elevated S1P level. (**A**) IB from the kidneys of WT and SPL^R222Q^ mice with and without added B6, showing reduced level of SPL protein only in R222Q mice with NA-B6 chow. (**B**–**E**) SPL activity in vital organs (kidney, lung, liver, and brain) of WT and SPL^R222Q^ mice, confirming drastically low activity of SPL in SPL^R222Q^ mice fed NA-B6 chow; *n* = 3. (**F**–**J**) Measurement of S1P levels in the plasma, kidney, lung, liver, and brain confirms higher levels of S1P in the organs and plasma of SPL^R222Q^ mice fed NA-B6 chow; *n* = 3. Data represent mean ± SD. *P ≤ 0.05, **P ≤ 0.01, ***P ≤ 0.001, and ****P ≤ 0.0001. A 2-tailed parametric *t* test was performed.

**Figure 4:**
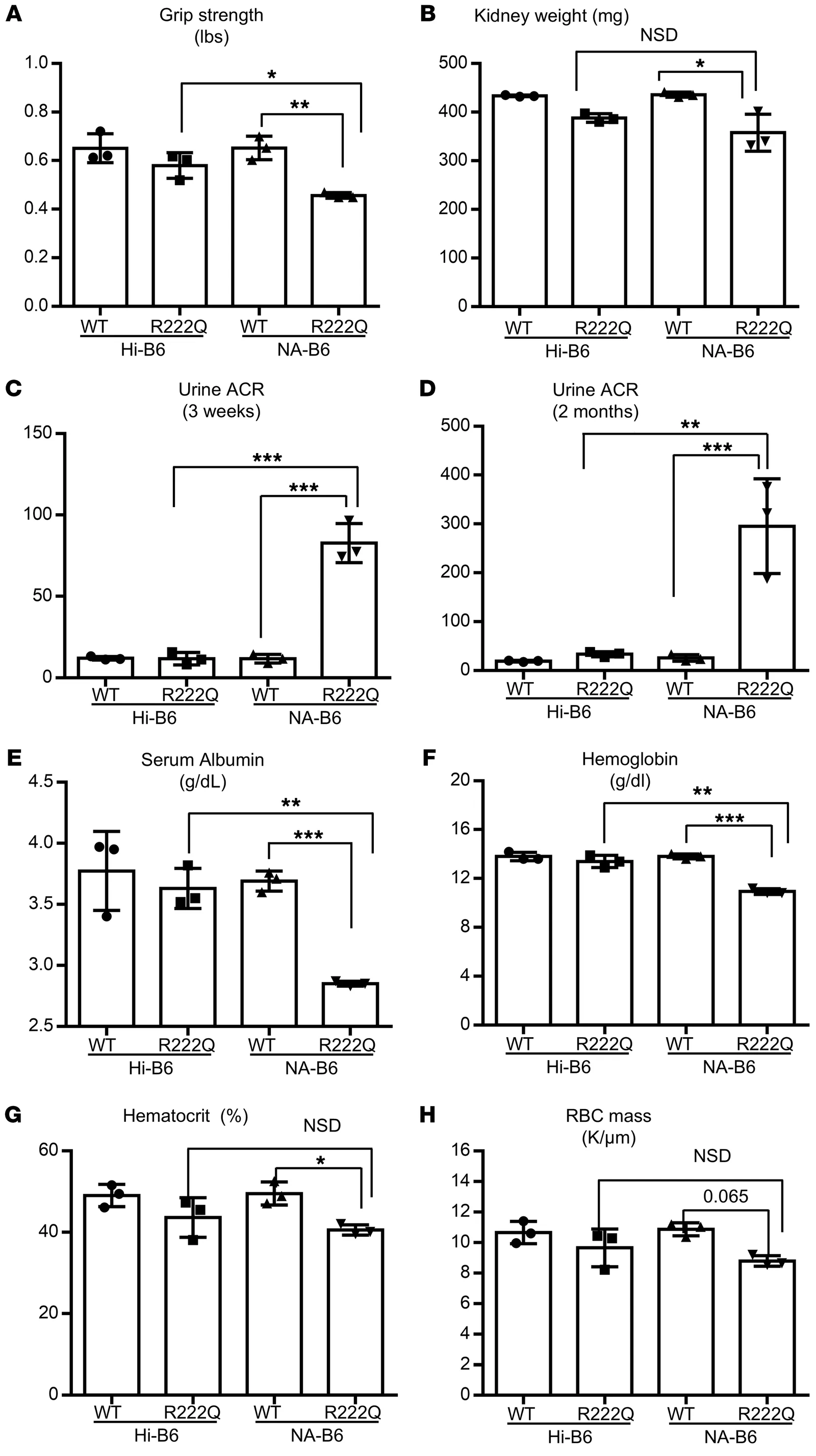
Pyridoxine supplementation ameliorates SPLIS features in SPL^R222Q^ mice. (**A**) Improvement in grip strength observed with B6 supplementation. (**B**) Kidney weight. (**C** and **D**) Urine ACR at 3- and 8-week time points, showing improvement in SPL^R222Q^ mice on Hi-B6 chow. (**E**) Serum albumin level. (**F**–**H**) RBC indices. Data represent mean ± SD. *P ≤ 0.05, **P ≤ 0.01, and ***P ≤ 0.001. NSD, no significant difference. One-way ANOVA with Bonferroni’s multiple comparisons was performed. *n* = 3 for each graph.

**Figure 5:**
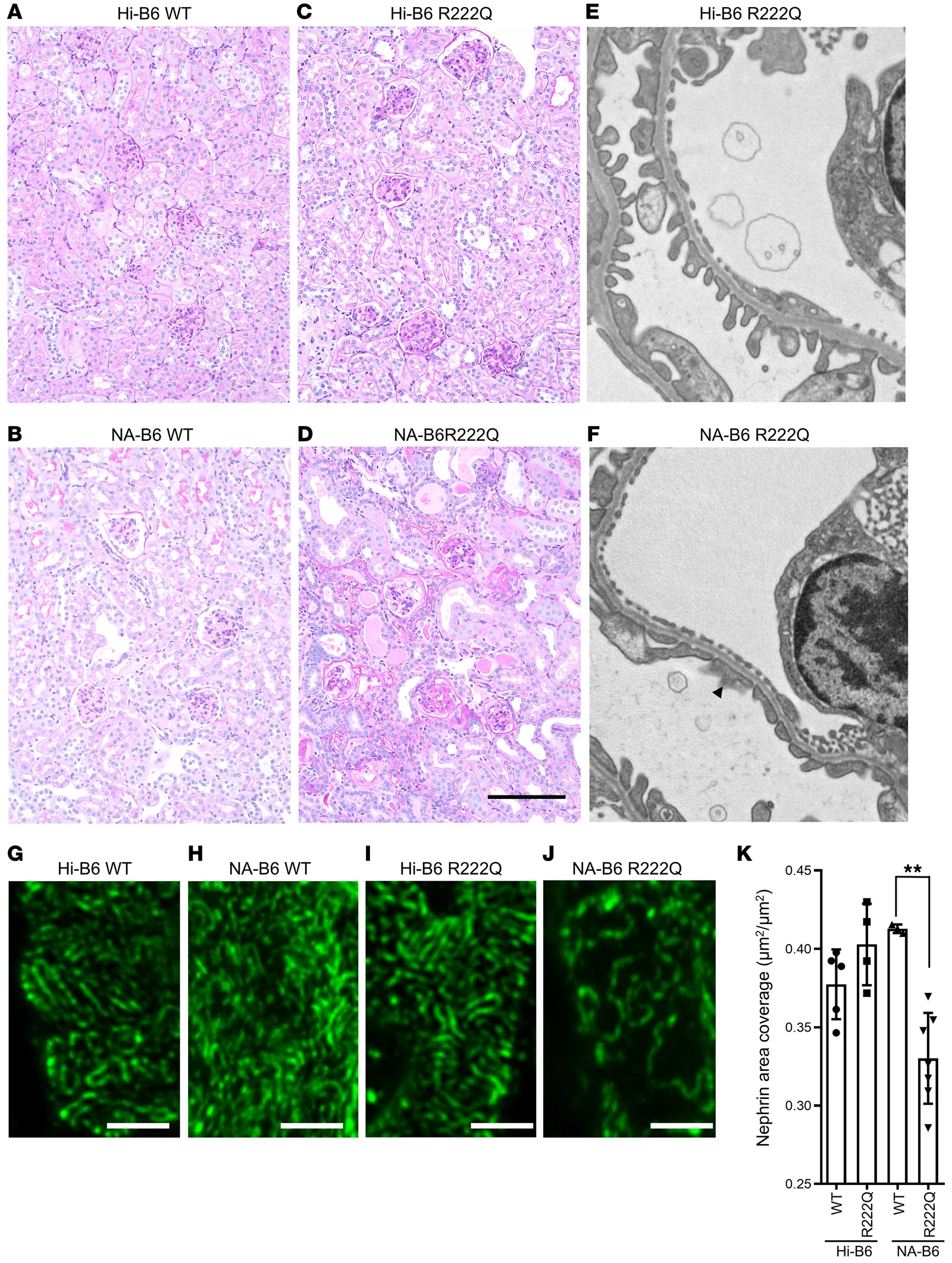
Addition of Hi-B6 in the chow prevented glomerulosclerosis, podocyte loss, and foot process effacement in SPL^R222Q^ mice. (**A**–**D**) PAS staining shows glomerulosclerosis in SPL^R222Q^ mice that were fed NA-B6 chow, while SPL^R222Q^ mice on Hi-B6 and WT mice on either diet exhibited normal morphology of glomeruli and tubules. Scale bar: 100 μm. (**E** and **F**) Transmission electron microscopy reveals podocyte effacement in SPL^R222Q^ mice that were fed NA-B6 chow compared with control mice on NA-B6 chow. (**G**–**J**) STED imaging reveals disrupted foot process morphology in SPL^R222Q^ mice fed NA-B6 chow compared with SPL^R222Q^ mice on Hi-B6 and WT mice on either diet. Scale bars: 1 μm. (**K**) Each point represents a separate glomerulus. In each analyzed glomerulus, 4–5 regions of interest were measured, and nephrin area and region of interest area were summed and presented as ratio. Hi-B6 WT, *n* = 5; Hi-B6 R222Q, *n* = 4; NA-B6 WT, *n* = 3; NA-B6 R222Q; *n* = 7. Average ROI area = 20 μm^2^. **P ≤ 0.01. Two-way ANOVA with Tukey’s post hoc analysis was performed.

**Figure 6:**
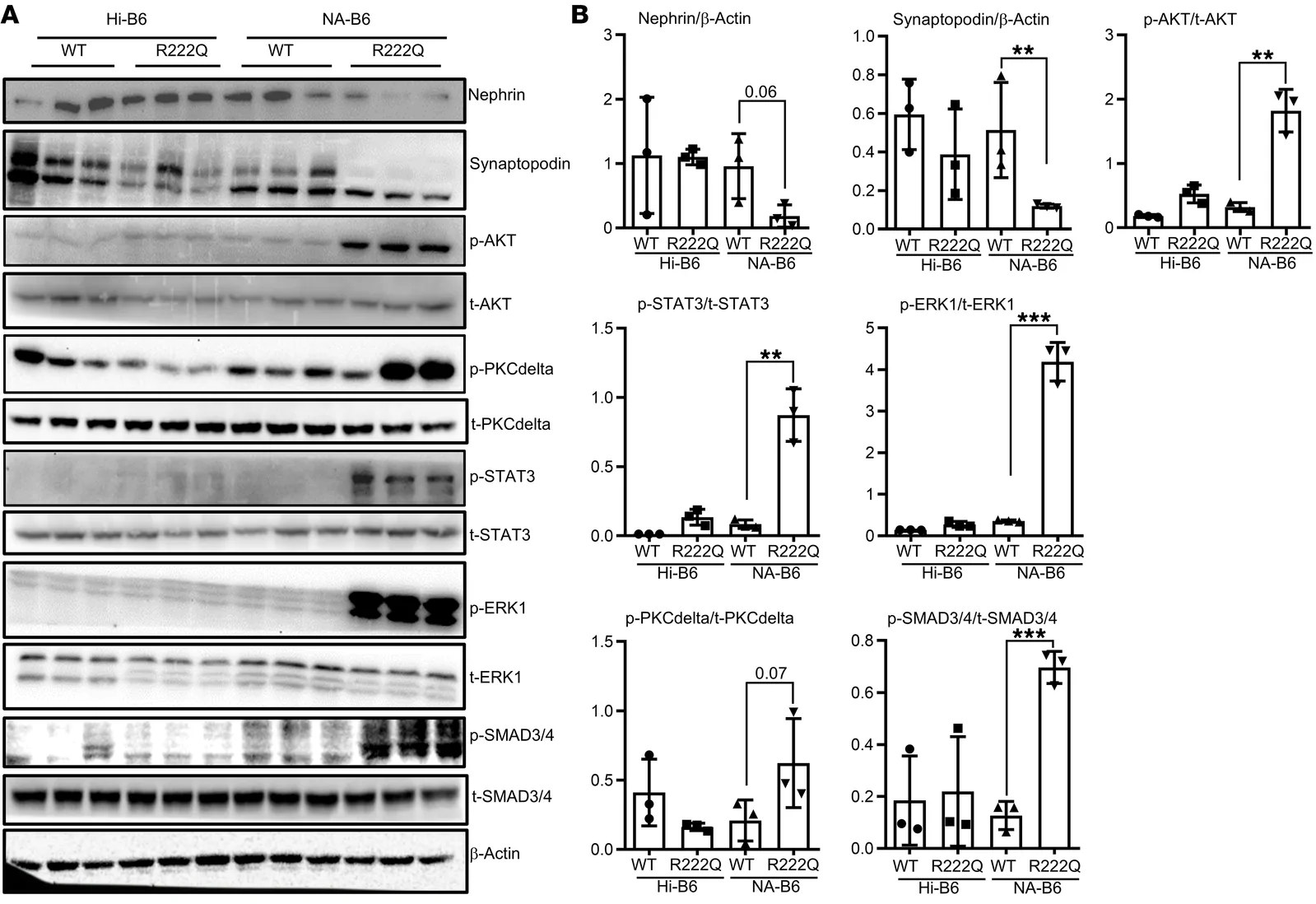
Pyridoxine supplementation prevented activation of stress signaling in SPL^R222Q^ mouse kidneys. (**A**) IB showing that podocyte markers nephrin and synaptopodin were reduced in the kidneys of SPL^R222Q^ mice on NA-B6 diet compared with SPL^R222Q^ mice on Hi-B6 and WT mice on either diet. Hyperactivation of p-AKT, p-PKCδ, p-STAT3, p-ERK1, and p-SMAD is observed in the kidneys of SPL^R222Q^ mice on NA-B6 chow. β-Actin was used as a loading control. (**B**) Quantification of corresponding IB. Data represent mean ± SD. **P ≤ 0.01 and ***P ≤ 0.001. A 2-tailed parametric *t* test was performed. *n* = 3 for each graph.

**Figure 7:**
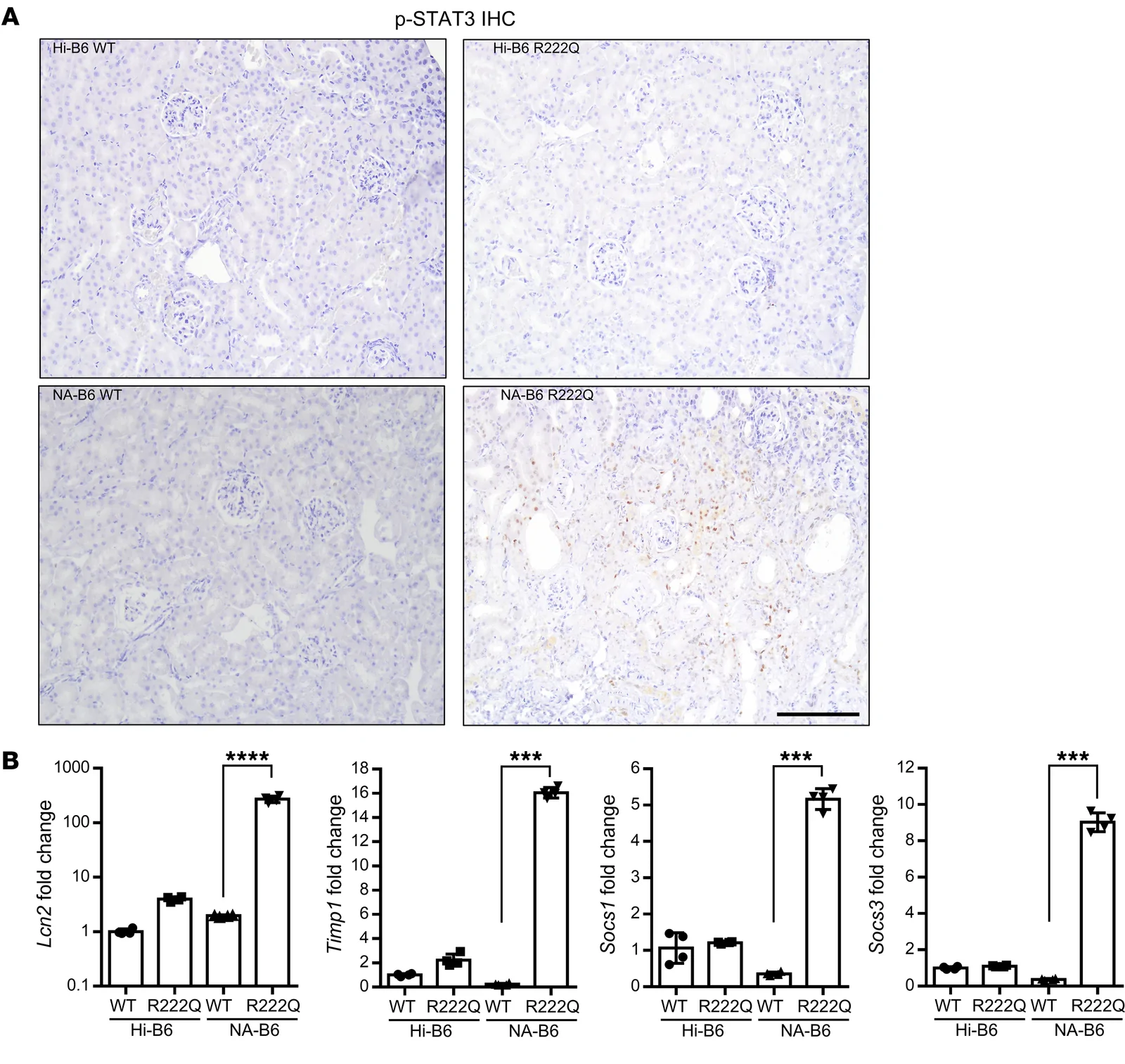
Low levels of pyridoxine cause activation of p-STAT3 and its target genes in the kidneys of SPL^R222Q^ mice. (**A**) p-STAT3 IHC staining displaying more staining in kidney sections of SPL^R222Q^ mice fed NA-B6 chow compared with SPL^R222Q^ mice on Hi-B6 and WT mice on either diet. Scale bar = 100 µm. (**B**) Relative expression of STAT3 target genes *Lcn2*, *Timp1*, *Socs1*, and *Socs3* (normalized with β-actin) in SPL^R222Q^ mice fed NA-B6 chow compared with SPL^R222Q^ mice on Hi-B6 and WT mice on either diet. Data represent mean ± SD. ***P ≤ 0.001 and ****P ≤ 0.0001. A 2-tailed parametric *t* test was performed. *n* = 3 for each graph.

**Figure 8:**
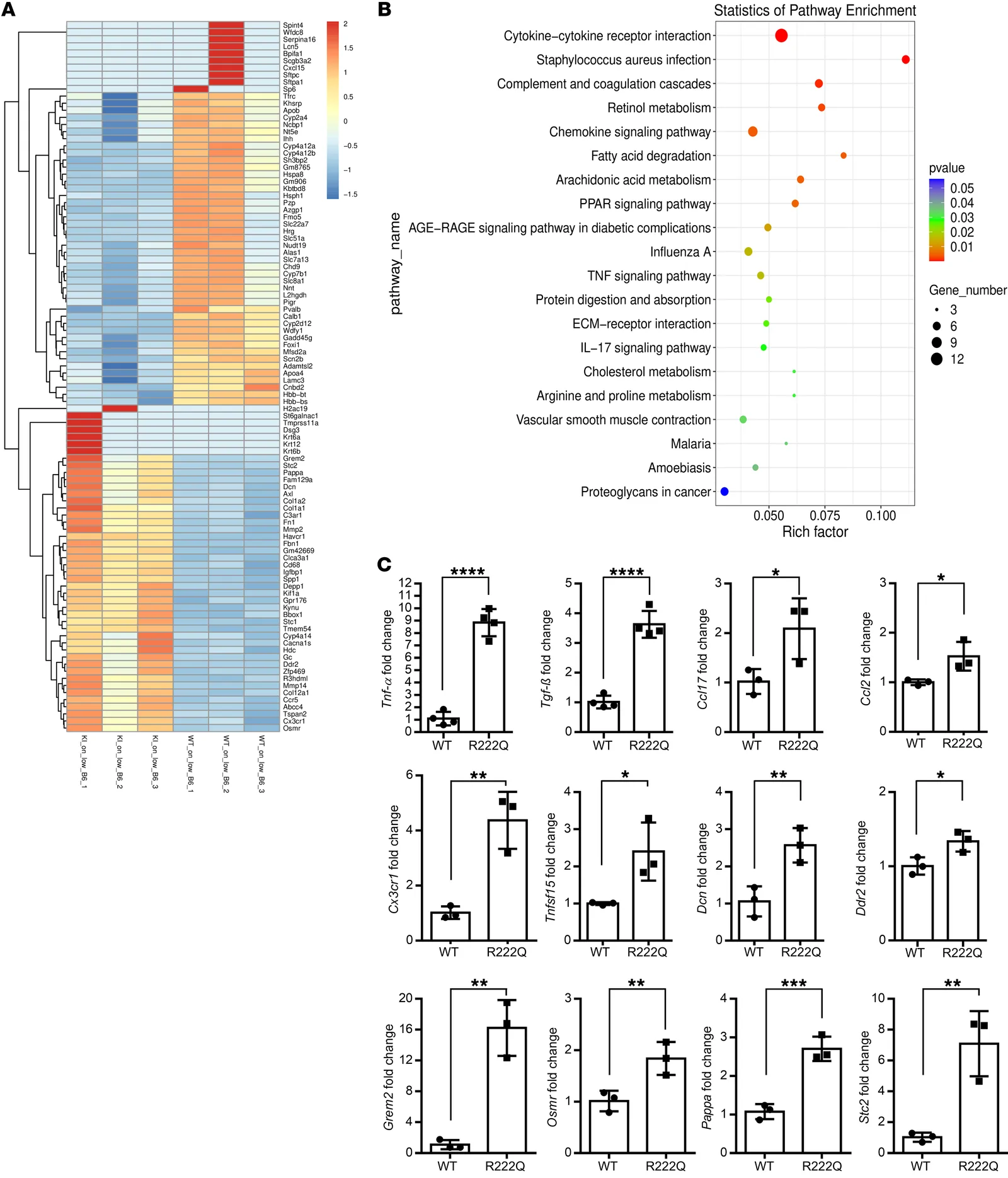
RNA-seq analysis and qPCR validation of target genes. (**A**) Heatmap displaying upregulation of chemokine, cytokine, extracellular matrix remodeling, and collagen-related genes in SPL^R222Q^ mice versus WT mice fed NA-B6 chow. (**B**) Statistics of pathway enrichment further confirm the aberrant regulation of chemokine, cytokine, immune, TNF-α, and other signaling pathways. (**C**) qPCR validation of target genes. At least 1–2 target genes were validated from each upregulated pathway. Data represent mean ± SD. *P ≤ 0.05, **P ≤ 0.01, ***P ≤ 0.001, and ****P ≤ 0.0001. A 2-tailed parametric *t* test was performed. *n* = 4 for *Tnf-α* and *Tgf-β*, and *n* = 3 for all other targets.

**Figure 9:**
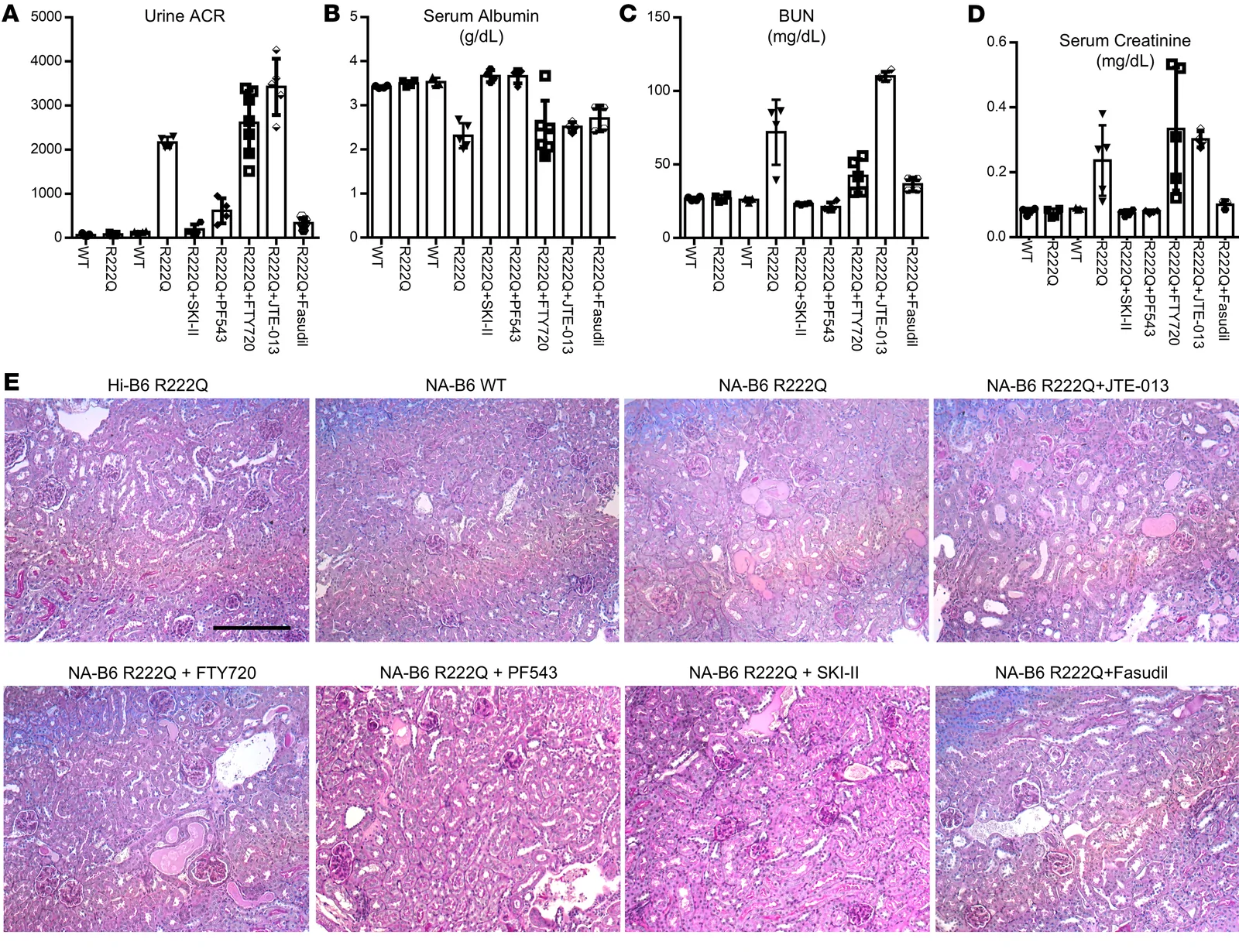
Inhibition of S1P production prevents SPLIS nephropathy. Parameters of kidney health were compared in WT and SPL^R222Q^ mice on Hi-B6 or NA-B6 diets versus SPL^R222Q^ mice on NA-B6, simultaneously treated with either the S1P receptor antagonist FTY720, JTE-013, the nonspecific SphK inhibitor SKI-II, the SphK1-specific inhibitor PF543, or the ROCK inhibitor fasudil. (**A**) Urine ACR. NA-B6-SPL^R222Q^ versus SPL^R222Q^ treated with SKI-II, SPL^R222Q^ treated with PF543, and SPL^R222Q^ treated with fasudil are statistically significant with a *P* value < 0.0001. NA-B6-SPL^R222Q^ versus SPL^R222Q^ treated with FTY720 and JTE-013 are not significantly different (NSD). *n* = 5 for Hi-B6 WT, NA-B6 SPL^R222Q+SKI-II^, NA-B6 SPL^R222Q+PF543^, NA-B6 SPL^R222Q+JTE-013^, and NA-B6 SPL^R222Q+fasudil^. *n* = 6 for NA-B6 SPL^R222Q^, *n* = 4 for NA-B6 WT, and *n* = 7 for NA-B6 SPL^R222Q+FTY720^. (**B**) Serum albumin. *P* < 0.0001 for NA-B6-SPL^R222Q^ versus SPL^R222Q^ treated with SKI-II and SPL^R222Q^ treated with PF543. *n* = 4 for Hi-B6 WT, NA-B6 SPL^R222Q+SKI-II^, NA-B6 SPL^R222Q+PF543^, NA-B6 SPL^R222Q+JTE-013^, and NA-B6 SPL^R222Q+fasudil^. *n* = 5 for NA-B6 SPL^R222Q^, *n* = 4 for NA-B6 WT, and *n* = 6 for NA-B6 SPL^R222Q+FTY720^. *P* = 0.086 for NA-B6-SPL^R222^ versus SPL^R222Q^ treated with fasudil, and NA-B6-SPL^R222Q^ versus SPL^R222Q^ treated with FTY720 and JTE-013 are NSD. (**C**) BUN. *n* = 4 for Hi-B6 WT, NA-B6 SPL^R222Q+SKI-II^, NA-B6 SPL^R222Q+PF543^, NA-B6 SPL^R222Q+JTE-013^, and NA-B6 SPL^R222Q+fasudil^. *n* = 4 for NA-B6 SPL^R222Q^, *n* = 3 for NA-B6 WT, and *n* = 5 for NA-B6 SPL^R222Q+FTY720^. NA-B6-SPL^R222Q^ versus SPL^R222Q^ treated with SKI-II, *P* = 0.004. SPL^R222Q^ versus SPL^R222Q^ treated with PF543, *P* = 0.0039. NA-B6-SPL^R222Q^ versus SPL^R222Q^ treated with FTY720, *P* = 0.034. NA-B6-SPL^R222Q^ versus SPL^R222Q^ treated with JTE-013, *P* = 0.014. NA-B6-SPL^R222Q^ versus SPL^R222Q^ treated with fasudil, *P* = 0.020. (**D**) Serum creatinine. *n* = 4 for Hi-B6 WT, NA-B6 SPL^R222Q+SKI-II^, NA-B6 SPL^R222Q+PF543^, and NA-B6 SPL^R222Q+JTE-013^, and *n* = 3 for NA-B6 SPL^R222Q+fasudil^. *n* = 5 for NA-B6 SPL^R222Q^, *n* = 3 for NA-B6 WT, and *n* = 5 for NA-B6 SPL^R222Q+FTY720^. NA-B6-SPL^R222Q^ versus SPL^R222Q^ treated with SKI-II, *P* = 0.02. SPL^R222Q^ versus SPL^R222Q^ treated with PF543, *P* = 0.03. NA-B6-SPL^R222Q^ versus SPL^R222Q^ treated with fasudil, *P* = 0.081. NA-B6-SPL^R222Q^ versus SPL^R222Q^ treated with FTY720 and JTE-013 are NSD. (**E**) PAS staining shows severe glomerulosclerosis in NA-B6-SPL^R222Q^ mice treated with FTY720 and JTE-013, while the disease phenotype was ameliorated in NA-B6-SPL^R222Q^ mice treated with SKI-II, PF543, and fasudil. Scale bar: 100 μm. Data represent mean ± SD. A 2-tailed parametric *t* test was performed.

## Version 1 09/24/2026

In-Press Preview

## Version 2 11/16/2026

Electronic publication
