# Supplementary material for: Pyridoxine supplementation ameliorates SGPL1 R222Q variant sphingosine phosphate lyase insufficiency syndrome in mice
Source: J Clin Invest. 2026 Sep 24;136(22):e206980. doi: 10.1172/JCI206980 (PMC13767628; doi:10.1172/JCI206980)
Supplement: Supplementary file 1 [file jci-136-206980-s106.pdf]

Supplementary Data Table of Contents

1. **Table S1.** Patient kidney functional data
2. **Table S2.** Quantification of glomerulosclerosis
3. **Table S3.** CBC analysis of WT mice treated with inhibitors
4. **Table S4.** Kidney function analysis of WT mice treated with inhibitors
5. **Table S5.** List of primers used for mutagenesis and qRT-PCR
6. **Figure S1.** Pyridoxine prevented disease progression in a patient with SPLIS-associated SPL^{R222Q} variant
7. **Figure S2.** PCA analysis and sample correlation
8. **Figure S3.** Heatmap analysis of WT on Hi-B6, WT on NA-B6, and SPL^{R222Q} on Hi-B6 chow
9. **Figure S4.** Overview analysis of whole kidney transcriptome RNA-seq
10. **Figure S5.** KEGG pathway showing involvement of cytokines in activating stress signals
11. **Figure S6.** Relative expression of a set of genes in the kidney of NA-B6-WT and NA-B6-SPL^{R222Q} mice treated with inhibitors
12. **Figure S7.** Immunoblotting for the downstream targets of FTY720 antagonist receptors in the whole kidney sample
13. **Pyridoxine calculations**

Table S1. Patient (IV:4) kidney functional data

Urine microalbumin (mg/L)	3.0
Spot urine creatinine (mg/dl)	157
Blood urea nitrogen (mg/dl)	9
Serum Creatinine (mg/dl)	0.5
eGFR	>90 ml/min/1.73
LFTs	Normal

Table S2. Quantification of glomerulosclerosis

Genotype	Total Glomeruli	Focal segmental glomerulosclerosis (%)	Global glomerulosclerosis (%)
WT Hi-B6 (n = 3)	241	0	0
WT NA-B6 (n =3)	175	0	0
SPL^{R222Q} Hi-B6 (n =3)	230	0	0
SPL^{R222Q} NA-B6 (n =3)	299	14.2	8.56

Table S3. CBC analysis of WT mice treated with inhibitors

Genotype (n = 4)	Absolute Lymphocyte (k/ul)	% Lymphocyte	RBS mass (M/ul)
WT-Hi-B6[a]	3.67± 1.06	74.00±1.47	7.97±0.17
W-NA-B6	1.94±0.14	70.92±6.0	8.77±0.61
WT-Hi-B6 + FTY720[b]	0.84±0.17	37.64±3.80	9.52±1.22
WT-NA-B6 + FTY720[c]	0.56±0.18	31.10±2.95	6.85±1.73
WT-Hi-B6 + PF543[d]	3.01±0.45	72.64±0.27	8.63±0.35
WT-NA-B6 + PF543[e]	2.34±0.32	66.91±2.28	9.10±0.18
WT-Hi-B6 + SKI-II[f]	2.22±0.23	69.59±1.81	8.63±0.11
WT-NA-B6 + SKI-II[g]	2.12±0.71	54.73±6.69	7.35±0.34
**p-values**			
WT-Hi-B6 vs WT-NA-B6	0.06	0.82	0.23
WT-Hi-B6 vs WT-Hi-B6 FTY720	0.003	4.6E-06	0.07
WT-NA-B6 vs WT-NA-B6 FTY720	4.85E-05	4.86E-05	0.21
WT-Hi-B6 vs WT-Hi-B6 PF543	0.35	0.16	0.02
WT-NA-B6 vs WT-NAB6 PF543	0.01	0.66	0.57
WT-Hi-B6 vs WT-Hi-B6 SKI-II	0.060	0.01	0.001
WT-NA-B6 vs WT-NA-B6 SKI-II	0.67	0.02	0.013

Table S4. Kidney function analysis of WT mice treated with inhibitors

Genotype	Urine ACR (n=4)	Serum Albumin (g/dL) (n = 3)	BUN (mg/dL) (n = 3)	Creatinine (mg/dL) (n = 3)
WT-Hi-B6	92.68±37.68	3.88±0.30	22.84±1.16	0.12±0.04
W-NA-B6	96.75±20.07	3.73±0.29	24.47±0.50	0.15±0.03
WT-Hi-B6 + FTY720	176.28±61.03	3.62±0.01	30.37±0.19	0.08±0.01
WT-NA-B6 + FTY720	249.39±94.83	3.64±0.03	32.4±0.33	0.07±0.004
WT-Hi-B6 + PF543	82.99±8.94	3.96±0.02	27.67±0.34	0.07±1.16
WT-NA-B6 + PF543	49.15±14.96	3.76±0.12	25.27±1.52	0.08±0.02
WT-Hi-B6 + SKI-II	53.17±8.71	3.42±0.31	38.27±2.78	0.07±0.003
WT-NA-B6 + SKI-II	103.62±23.32	3.04±0.02	34.10±0.36	0.08±0.01
WT-Hi-B6 + Fasudil	104.90±52.39	3.89±0.09	28.41±3.72	0.13±0.04
WT-NA-B6 + Fasudil	103.23±34.81	3.71±0.14	25.86±7.11	0.11±0.01
WT-Hi-B6 + JTE-013	230.79±40.51	2.28±0.04	45.63±1.03	0.09±0.004
WT-NA-B6 + JTE-013	256.68±53.17	3.26±0.31	21.17±2.79	0.08±0.02
**p-values**				
WT-Hi-B6 vs WT-NA-B6	0.87	0.6483	0.1421	0.4977
WT-Hi-B6 vs WT-Hi-B6 FTY720	0.034	0.2963	0.0008	0.2929
WT-NA-B6 vs WT-NA-B6 FTY720	0.090	0.6734	0.0001	0.0276
WT-Hi-B6 vs WT-Hi-B6 PF543	0.68	0.7206	0.0409	0.1884
WT-NA-B6 vs WT-NAB6 PF543	0.017	0.8987	0.5178	0.0738
WT-Hi-B6 vs WT-Hi-B6 SKI-II	0.71	0.2099	0.0019	0.1804
WT-NA-B6 vs WT-NA-B6 SKI-II	0.13	0.0279	0.0001	0.0431

WT-Hi-B6 vs WT-Hi-B6 Fasudil	0.75	0.9669	0.1134	0.8545
WT-NA-B6 vs WT-NA-B6 Fasudil	0.79	0.9122	0.7961	0.2391
WT-Hi-B6 vs WT-Hi-B6 JTE	0.003	0.1120	0.4784	0.3383
WT-NA-B6 vs WT-NA-B6 JTE	0.005	0.0022	0.0001	0.0685

Table S5. List of primers used for mutagenesis and qRT-PCR.

**Primer name**	**Sequence (5'-3')**
h-*SGPL1* -WT-F	GGAATTCCATATGAATAAAACTAAGGATGATATCAGCAAGAATATG
h-*SGPL1* -WT-R	ATATATCTCGAGTTAATGATGATGGTGATGATGACCGCTACCGTGAGGTTTA GGGCTTCCATTCATCTG
h-*SGPL1* - R222Q -F	AAAGCCTACCAGGATCTTGCGTTTGAGAAGGG
h-*SGPL1* - R222Q -R	AACGCAAGATCGTCGTAGGCTTTGCATGCCAT
h-*SGPL1* - R222 W-F	AAAGCCTACTGGGATCTTGCGTTTGAGAAGGG
h-*SGPL1* - R222 W-R	AACGCAAGATCCCAGTAGGCTTTGCATGCCAT
m*Lcn2* -F	TGGCCCTGAGTGTCATGTG
m*Lcn2* -R	CTCTTGTAGCTCATAGATGGTGC

m*Timp 1*-F	GCAACTCGGACCTGGTCATAA
m*Timp 1*-R	CGGCCCGTGATGAGAAACT
m*Socs 1*-F	CTGCGGCTTCTATTGGGGAC
m*Socs 1*-R	AAAAGGCAGTCGAAGGTCTCG
m*Socs 3*-F	CCCTTGCAGTTCTAAGTTCAACA
m*Socs 3*-R	ACCTTTGACAAGCGGACTCTC
m*Tnf-α*-F	CAGGCGGTGCCTATGTCTC
m*Tnf-α*-R	CGATCACCCCGAAGTTCAGTAG
m*Tgfβ*-F	CCACCTGCAAGACCATCGAC
m*Tgfβ*-R	CTGGCGAGCCTTAGTTTGGAC
m*Ccl2*-F	TGCCCTAAGGTCTTCAGCAC
m*Ccl2*-R	AAGGCATCACAGTCCGAGTC
m*Ccl17*-F	AAGCTCATCTGTGCAGACCC
m*Ccl17*-R	CAGGGACTTCTGCTCTGTGG
m*Cx3cr1*-F	GCCTGTTATTTGGGCGACAT
m*Cx3cr1*-R	ACAAAGAGCAGGTCGCTCAA

m*Tnfsf15*-F	TGTGTGGTGACATCAGTCGG
m*Tnfsf15*-R	TCTTCCAAGGAGAACGTGGC
m*Dcn*-F	CTTCCTTCTGGCACAAGTCT
m*Dcn*-R	CAGAACACTGCACCACTCGA
m*Ddr2*-F	GGCTGGACTCTGAAGAAGGA
m*Ddr2*-R	CCATCCCGACTGTAGTTGAT
m*Grem2*-F	TGGTGGCTGTGCTGGTAAAG
m*Grem2*-R	TTGAGGTACTTGCGCTCGGT
m*Osmr*-F	GGACACGAAGAGGTCAAGGA
m*Osmr*-R	ATCATCTGAGGTGATGGTGG
m*Pappa*-F	ACAGCCCTGTAAATCCAGTG
m*Pappa*-R	CACTAGAGTCCCAATCACTGG
m*Stc2*-F	CATCAAGGATGCCCTGAGGT
m*Stc2*-R	TCACACCGACGTTCTCCTGG
m-B-Actin-F	GGCTGTATTCCCCTCCATCG

m-B-Actin-R	CCAGTTGGTAACAATGCCATGT

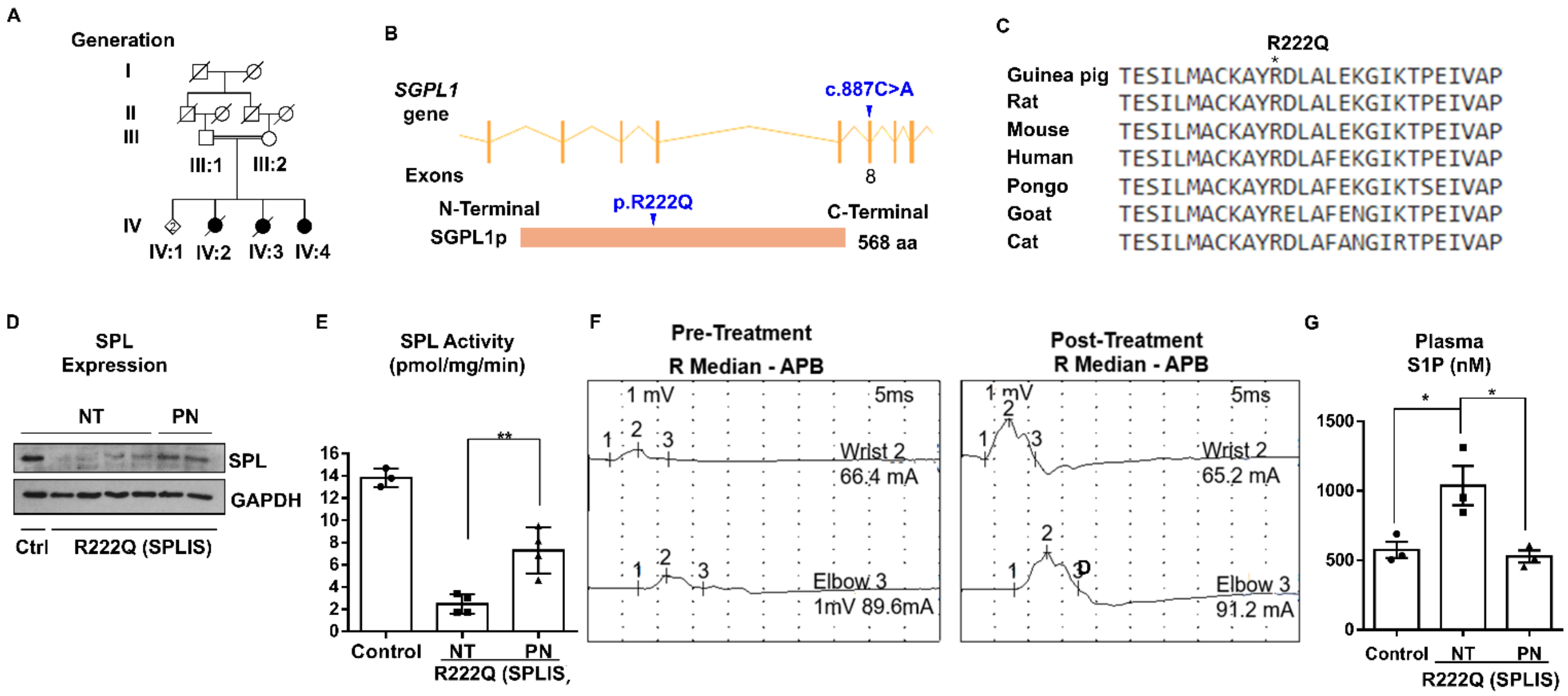

Figure S1. Pyridoxine prevented disease progression in a patient with SPLIS-associated SPL^{R222Q} variant. (A) An extended consanguineous pedigree showed the one alive female patient (IV:4) with SPLIS-related symptoms. (B) *SGPL1* missense variant (c.887C>A) in exon 8 was supposed to cause a change in protein folding, resulting in an unstable SPL protein. (C) Sequence alignment of the region containing part of SPL amino acid sequence in guinea pig, rat, mouse, human, pongo, goat, and cat showing highly conserved nature of arginine. (D) SPL expression was measured by IB of whole cell extracts of fibroblasts from a healthy control (Ctrl) propagated for 24h in medium with no added pyridoxine, i.e., no treatment (NT), versus fibroblasts from a patient homozygous for the SPL^{R222Q} variant (R222Q SPLIS) propagated in medium with no added PN for 24h followed by no further treatment (NT) or followed by propagation in medium containing 100 µM PN for 72h. GAPDH is a loading control. (E) Treatment of SPL^{R222Q} variant skin fibroblasts with pyridoxine increased SPL activity to ~25% of control fibroblasts. Untreated vs PN, $p < 0.005$. (E) Improvement in motor nerve conduction of the patient (IV:4) in post-B6 treatment. (F) Plasma S1P in the patient (IV:4) is reduced after two months of PN treatment. Aged match untreated vs post-B6 plasma S1P, $p < 0.04$. N = 3 for figure D, E and G. Data represents mean ± SD. 2-tailed parametric *t* test was performed.

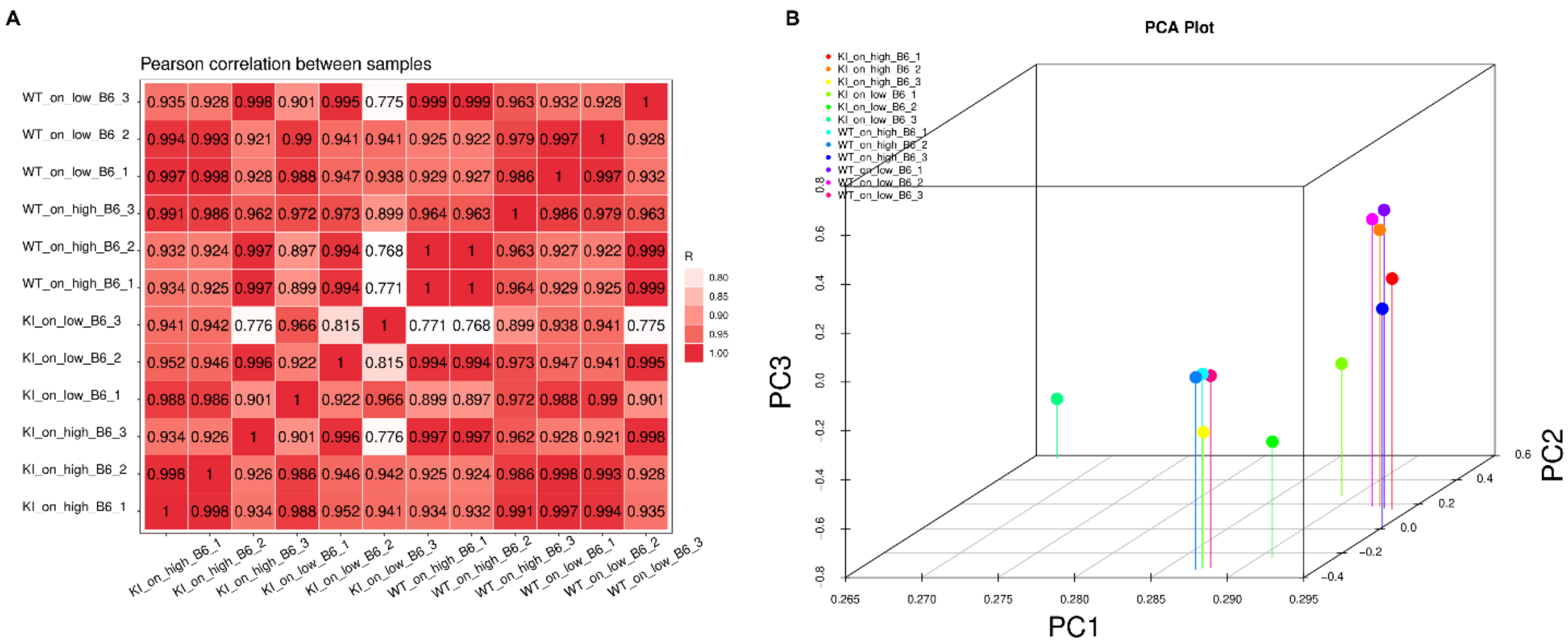

Figure S2. PCA analysis and sample correlation. (A) Pearson correlation is used to assess the linear relationship and similarity between different samples. (B) Principal component analysis (PCA) was used to simplify complex gene expression data, identify patterns, and visualize relationships between samples.

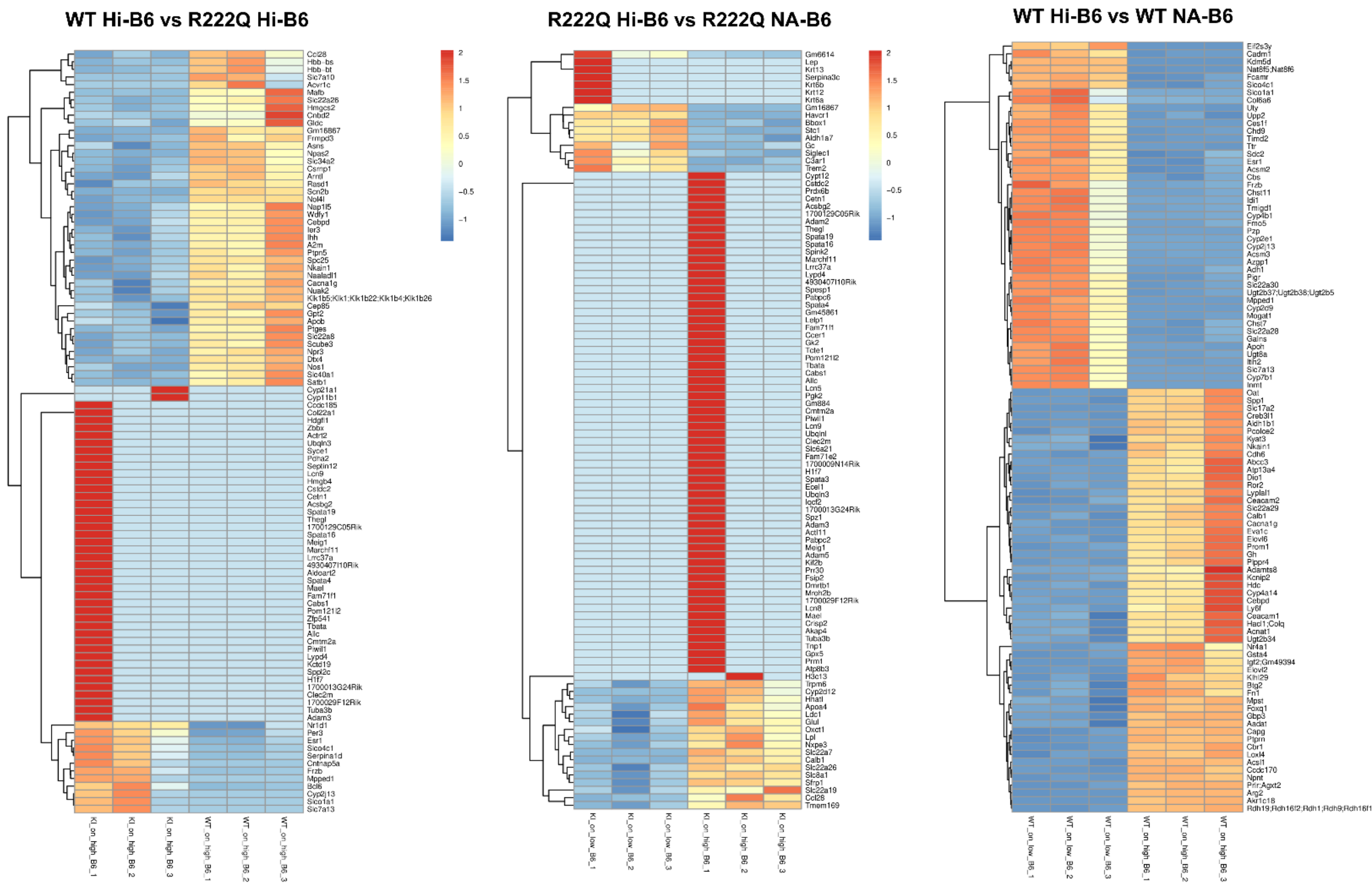

Figure S3. Heatmap analysis of WT on Hi-B6, WT on NA-B6, SPL^{R222Q} on Hi-B6 chow. We excluded those genes that were common between the WT Hi-B6 vs the WT NA-B6 group to avoid the effect of low B6.

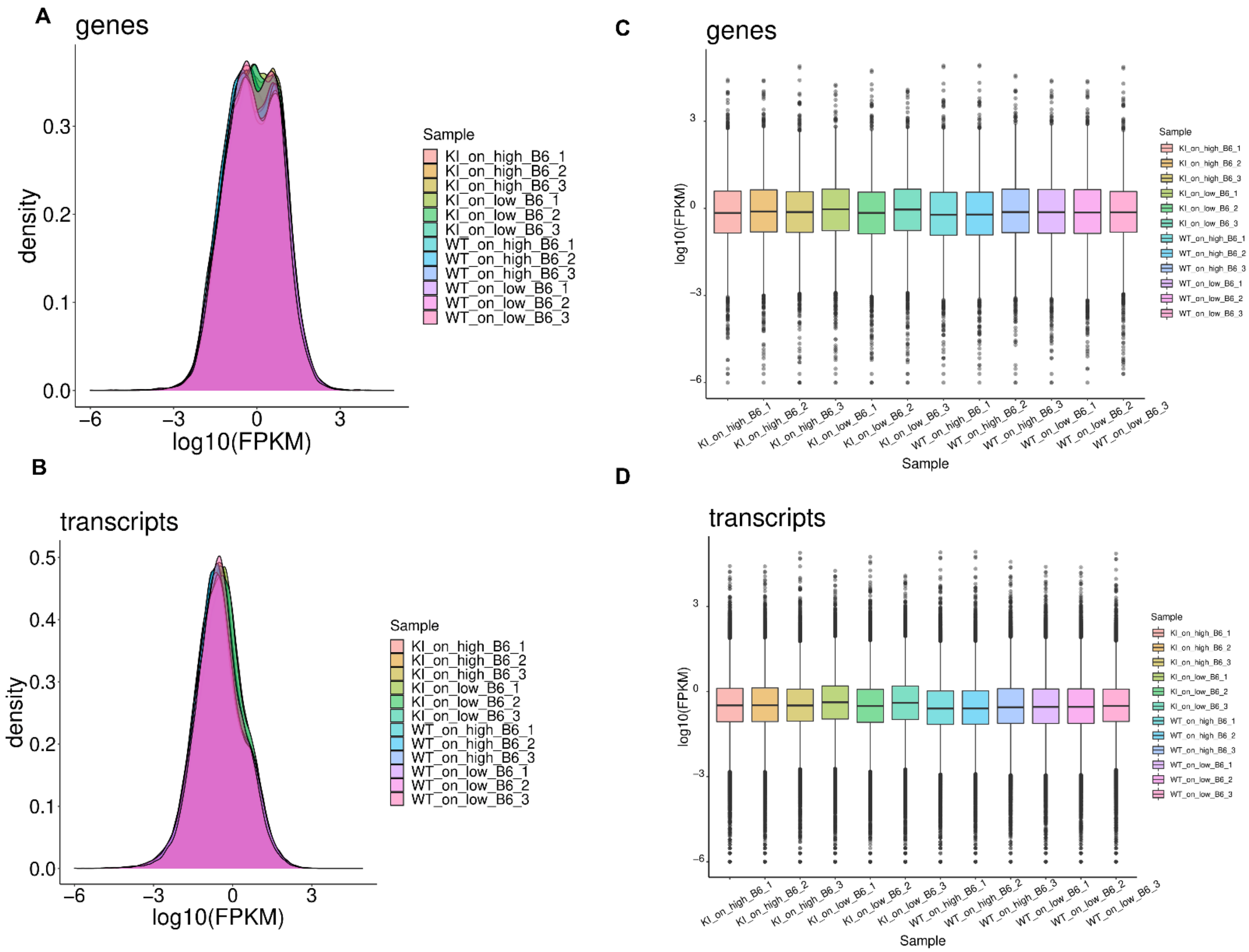

Figure S4. Overview analysis of whole kidney transcriptome RNA-seq. (A-D) Gene and transcript density levels (A&B) and boxplot expression (C&D) in different groups of samples were estimated by StringTie using FPKM (total_exon_fragments/mapped_reads(millions)×exon_length(kB)]). (E) Statistics of the mapped region. (F) Statistics of differentially expressed genes in each group. (G) Statistics of differentially expressed transcripts in each group.

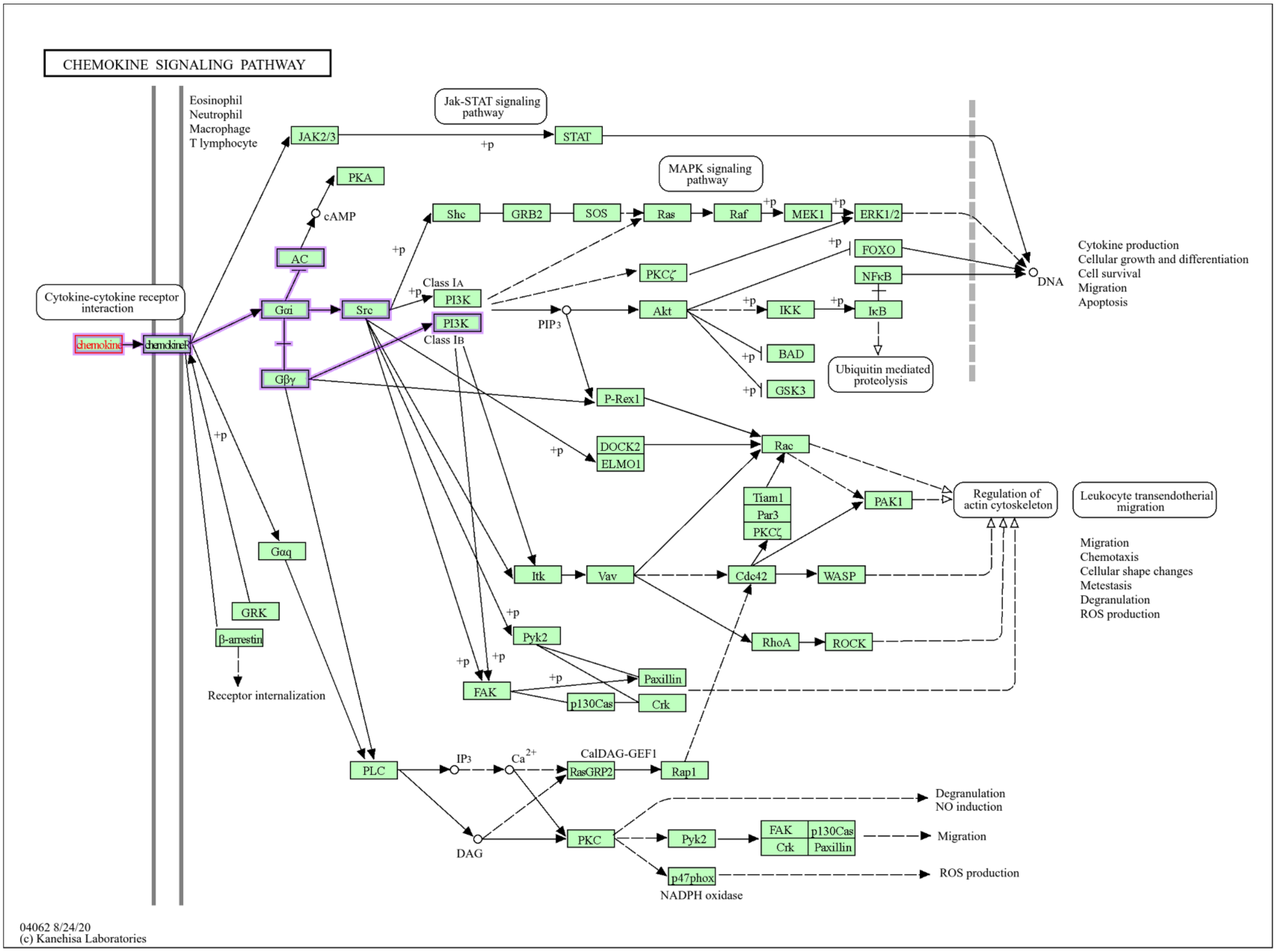

Figure S5. KEGG pathway showing the involvement of cytokines in activating stress signals.

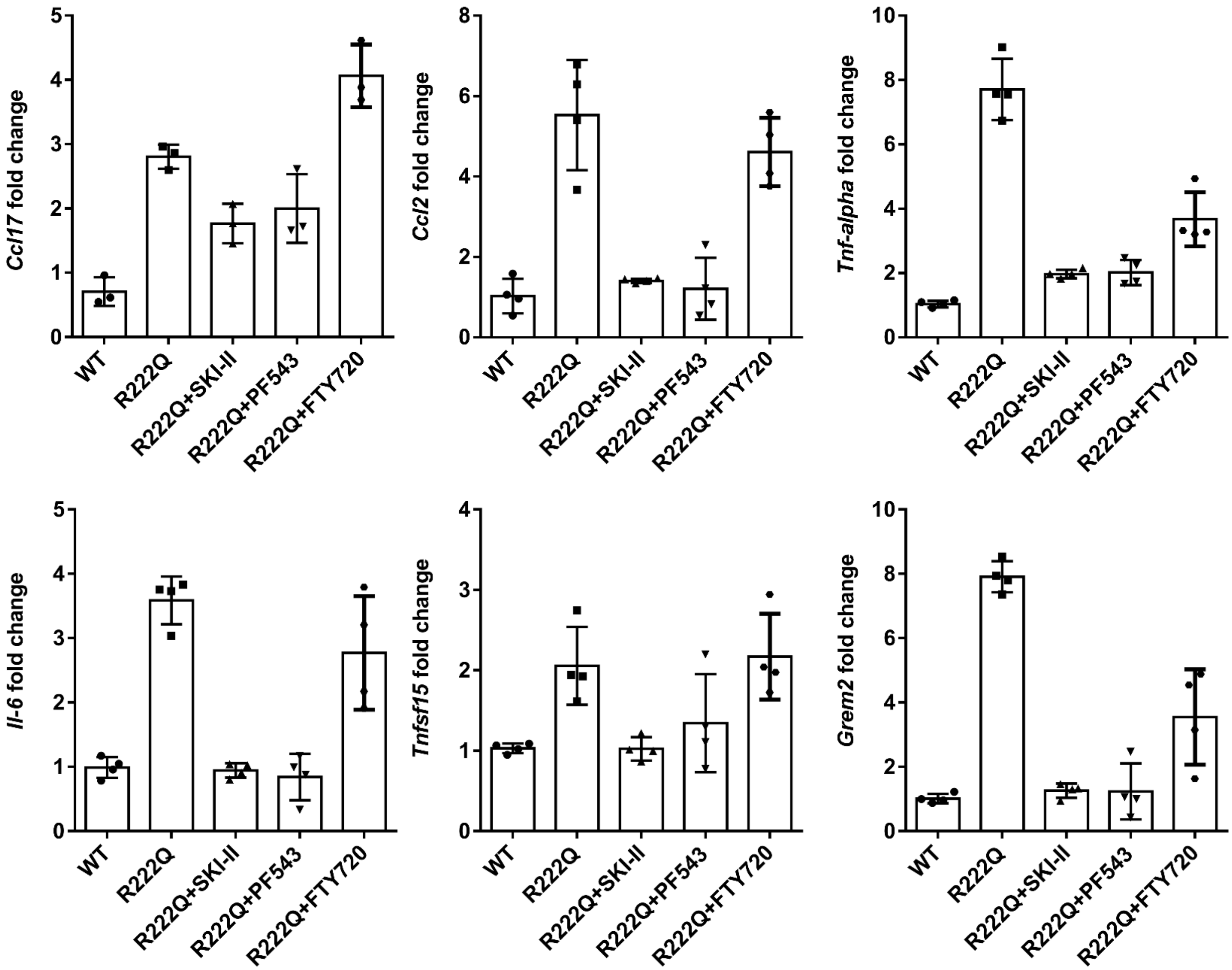

Figure S6. Relative expression of a set of genes in the kidney of NA-B6-WT and NA-B6-SPL^{R222Q} mice treated with inhibitors. Relative expression of target genes *Ccl17*, *Ccl2*, *Tnf-alpha*, *Il-6*, *Tnfsf15* and *Grem2* (normalized with b-actin) in NA-B6-SPL^{R222Q} mice treated with FTY720, SKI-II, PF543, and control mice. (n = 4). For *ccl17*, NA-B6-WT vs NA-B6-SPL^{R222Q}, p>0.0001, NA-B6-SPL^{R222Q} vs NA-B6-SPL$^{R222Q+FTY720}$ and NA-B6-SPL$^{R222Q+SKI-II}$, p >0.0009, while NA-B6-SPL^{R222Q} vs NA-B6-SPL$^{R222Q+PF543}$, p = 0.0042. For *ccl2*, NA-B6-WT vs NA-B6-SPL^{R222Q}, p = 0.0008, NA-B6-SPL^{R222Q} vs NA-B6-SPL$^{R222Q+FTY720}$ are NSD, while NA-B6-SPL^{R222Q} vs NA-B6-SPL$^{R222Q+PF543}$ and NA-B6-SPL$^{R222Q+SKI-II}$, p = 0.001. For *Tnf-alpha*, NA-B6-WT vs NA-B6-SPL^{R222Q}, p = 0.00001, while NA-B6-SPL^{R222Q} vs all NA-B6-SPL^{R222Q} treated group, p = 0.0001. For *Il-6*, NA-B6-WT vs NA-B6-SPL^{R222Q}, p = 0.00001, NA-B6-SPL^{R222Q} vs NA-B6-SPL$^{R222Q+FTY720}$ are NSD, while NA-B6-SPL^{R222Q} vs NA-B6-SPL$^{R222Q+PF543}$

and NA-B6-SPL$^{R222Q+SKI-II}$, p >0.0001. For *Tnfsf15*, NA-B6-WT vs NA-B6-SPL^{R222Q}, p = 0.005, NA-B6-SPL^{R222Q} vs NA-B6-SPL$^{R222Q+FTY720}$ and NA-B6-SPL$^{R222Q+PF543}$ are NSD, while NA-B6SPL^{R222Q} vs NA-B6-SPL$^{R222Q+PF543}$ p = 0.006. For *Grem2*, NA-B6-WT vs NA-B6-SPL^{R222Q}, p >0.0001, NA-B6-SPL^{R222Q} vs all NA-B6-SPL^{R222Q} treated group, p >0.0001. Data represents mean ± SD. 2-tailed parametric *t* test was performed.

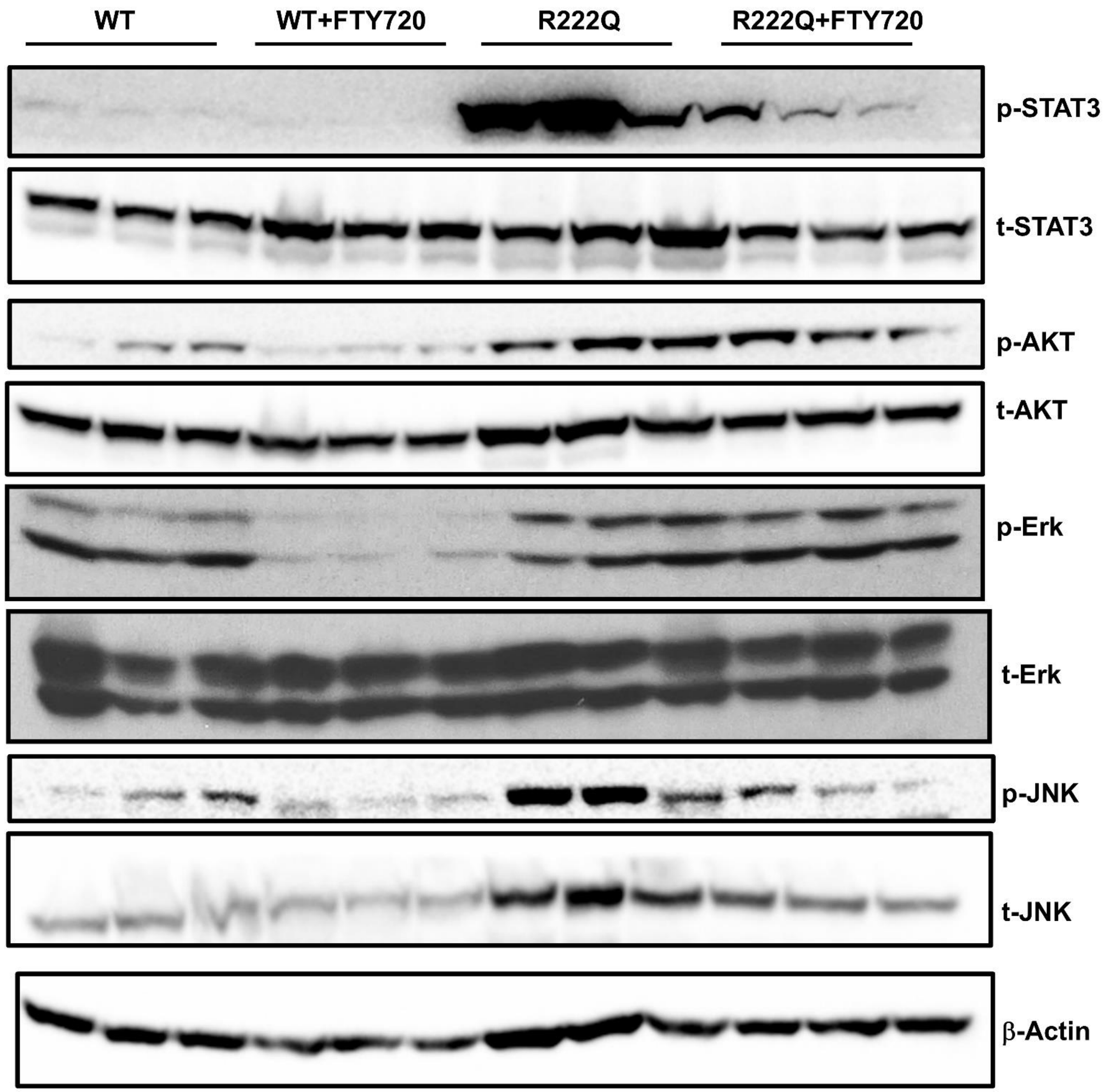

Figure S7 Immunoblotting for the downstream targets of FTY720 antagonist receptors in the whole kidney sample. Western blotting confirmed that the downregulation of downstream targets (ERK1, STAT3, AKT, and JNK) of key S1P receptors, which are antagonized by FTY720 in the kidney of NA-B6-WT and NA-B6-SPL^{R222Q} mice. N = 3.

Pyridoxine calculations

NA-B6 diet: NA-B6 diet with 2 ppm pyridoxine = 2 mg/kg chow. This translates to 10 micrograms of pyridoxine per day assuming a mouse is eating 5 g chow a day, or 0.5 mg/kg/d, which is about half of normal recommendation of 1 mg/kg/d for non-pregnant mice.

Hi-B6 diet: Based on the pyridoxine content of regular laboratory mouse chow and an estimated intake of 5g chow/day, we calculated that Hi-B6 diet resulted in 6-7 mg/kg/d pyridoxine, i.e., 6 to 7-fold higher than the minimum daily requirements.

Regarding the relationship of mouse and human B6 intake, recommendations for daily human vitamin B6 intake vary substantially by age but are between 0.1-1.0 mg per day. Assuming a 50 kg adult takes in 0.5 mg = 500 micrograms intake, that is roughly 10 micrograms/kg. High dose pyridoxine therapy is vastly higher than this, translating to between 75 and 500 times minimum daily requirements. Unfortunately, B6 intake is not equivalent between mice and humans. When scaled to body weight, mice require a substantially higher concentration of B6 relative to their mass — a common pattern with water-soluble vitamins in smaller mammals, which have faster metabolisms.
