# Supplementary material for: Pyridoxine supplementation ameliorates SGPL1 R222Q variant sphingosine phosphate lyase insufficiency syndrome in mice
Source: J Clin Invest. 2026 Sep 24;136(22):e206980. doi: 10.1172/JCI206980 (PMC13767628; doi:10.1172/JCI206980)

Figure 1A

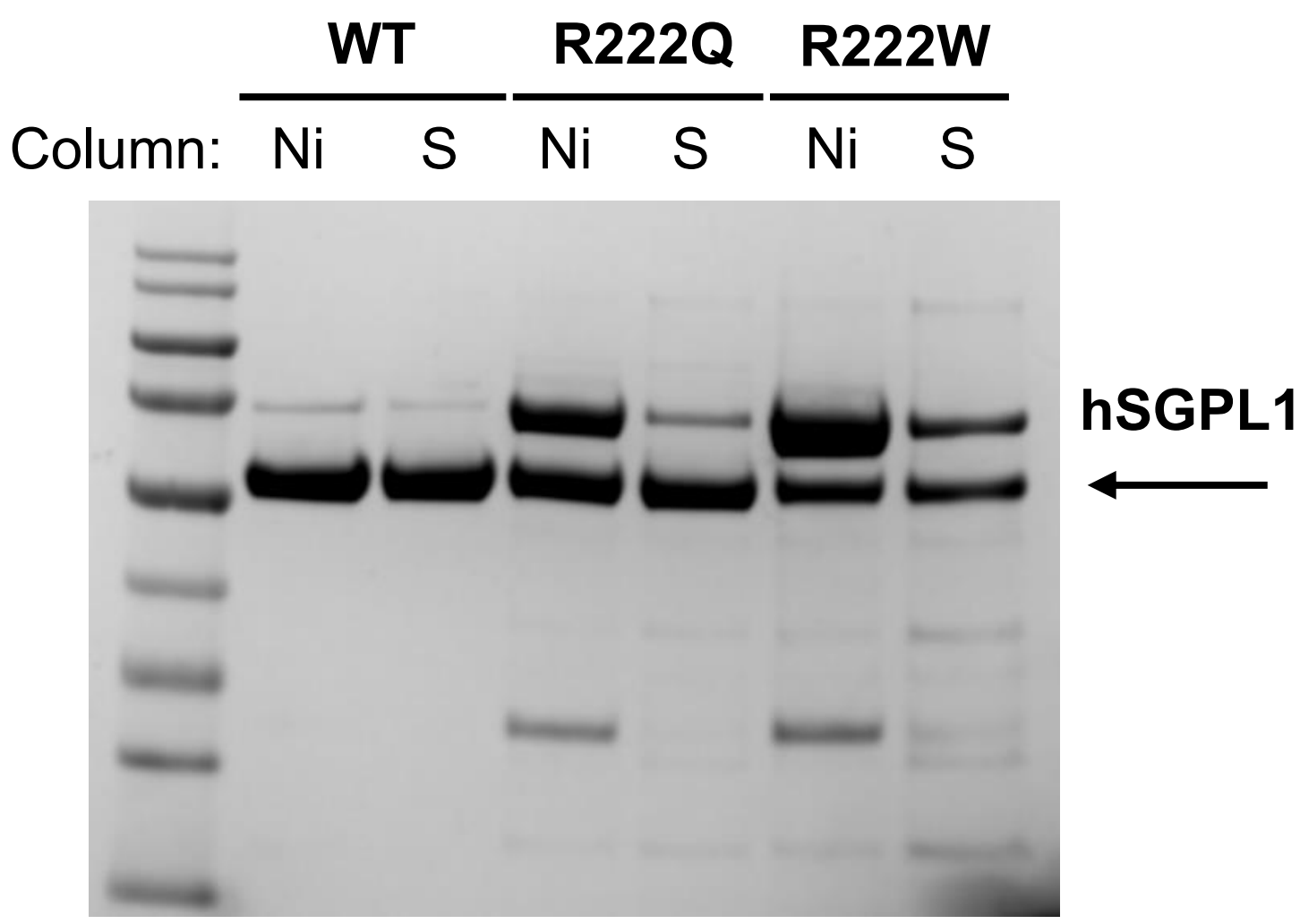

Figure 3 A

mSPL

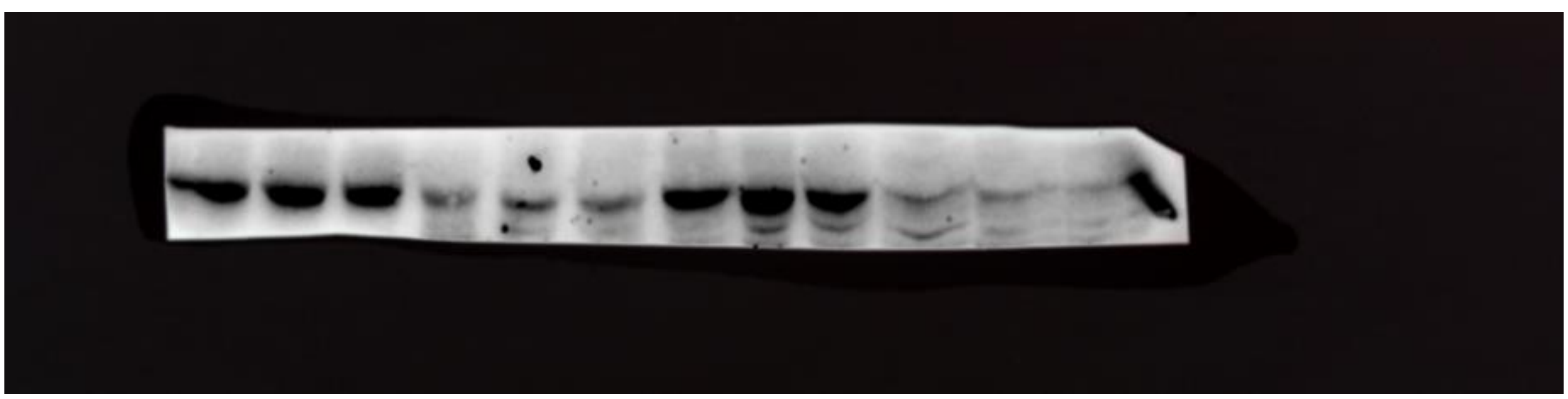

B-Actin

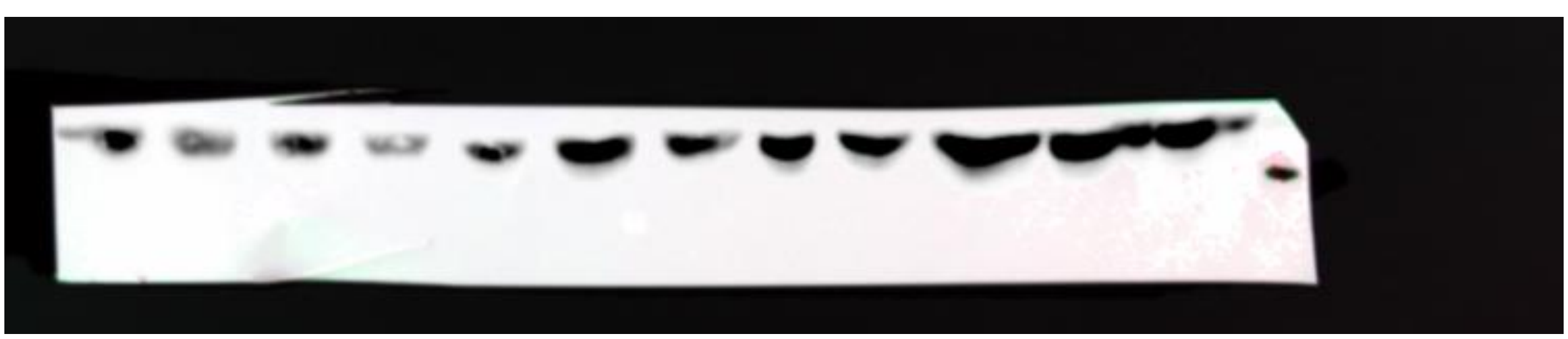

Figure 6

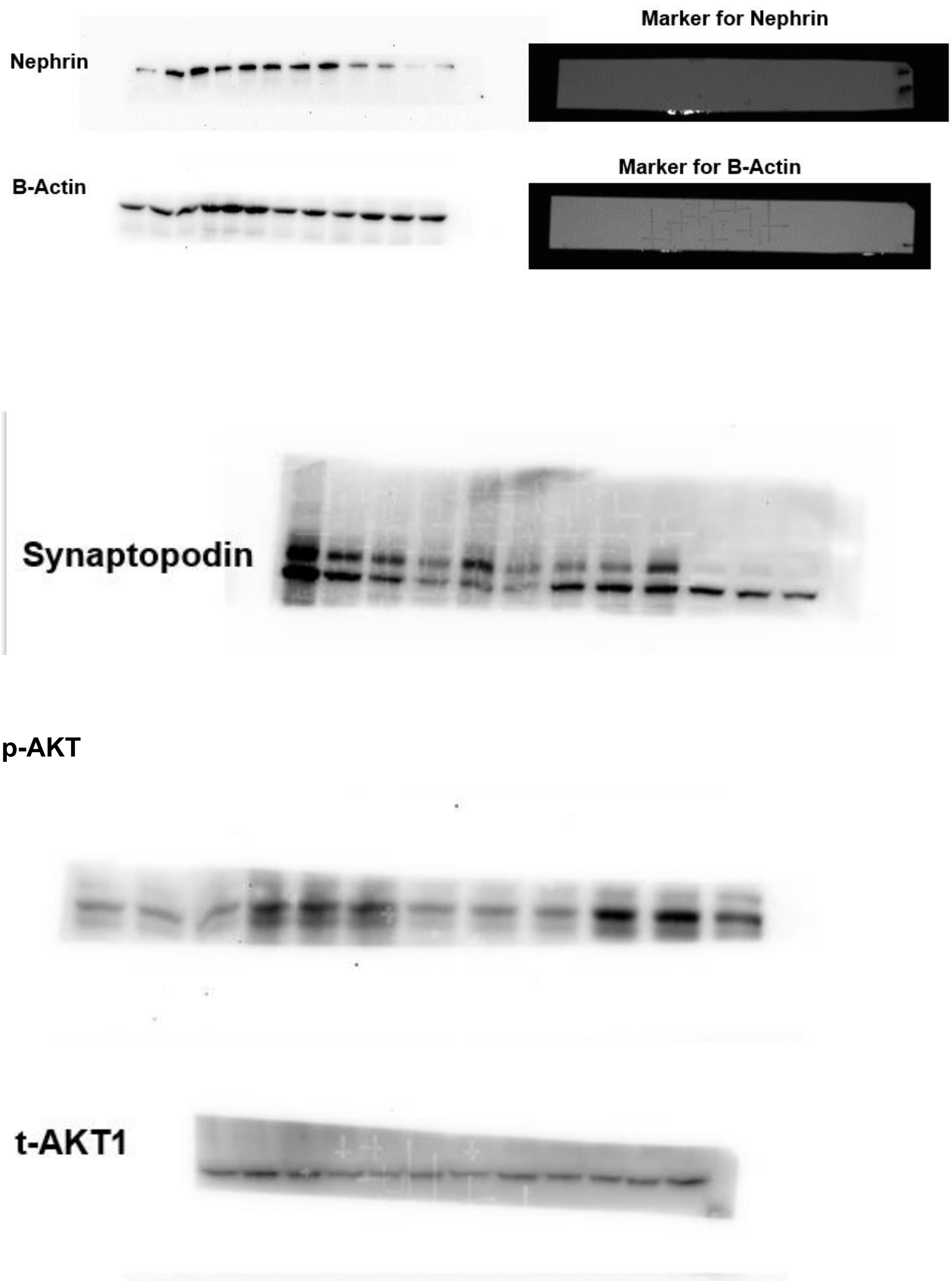
Nephrin
Marker for Nephrin
B-Actin
Marker for B-Actin
Synaptopodin
p-AKT
t-AKT1

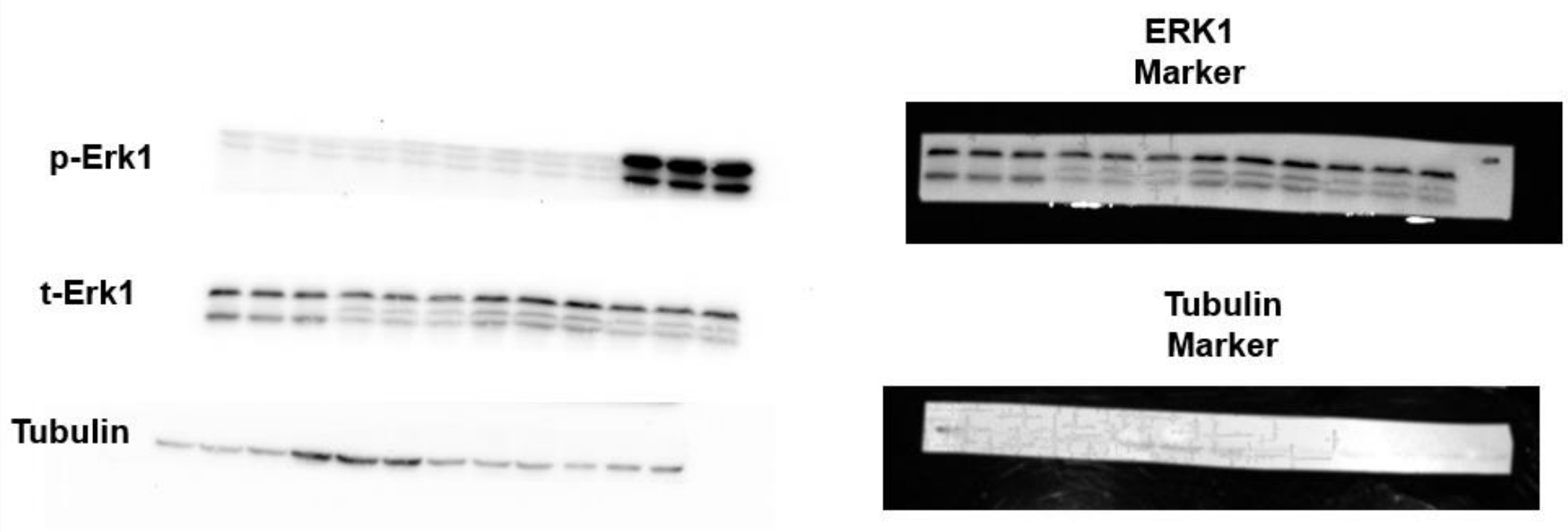
ERK1 Marker
p-Erk1
t-Erk1
Tubulin Marker
Tubulin

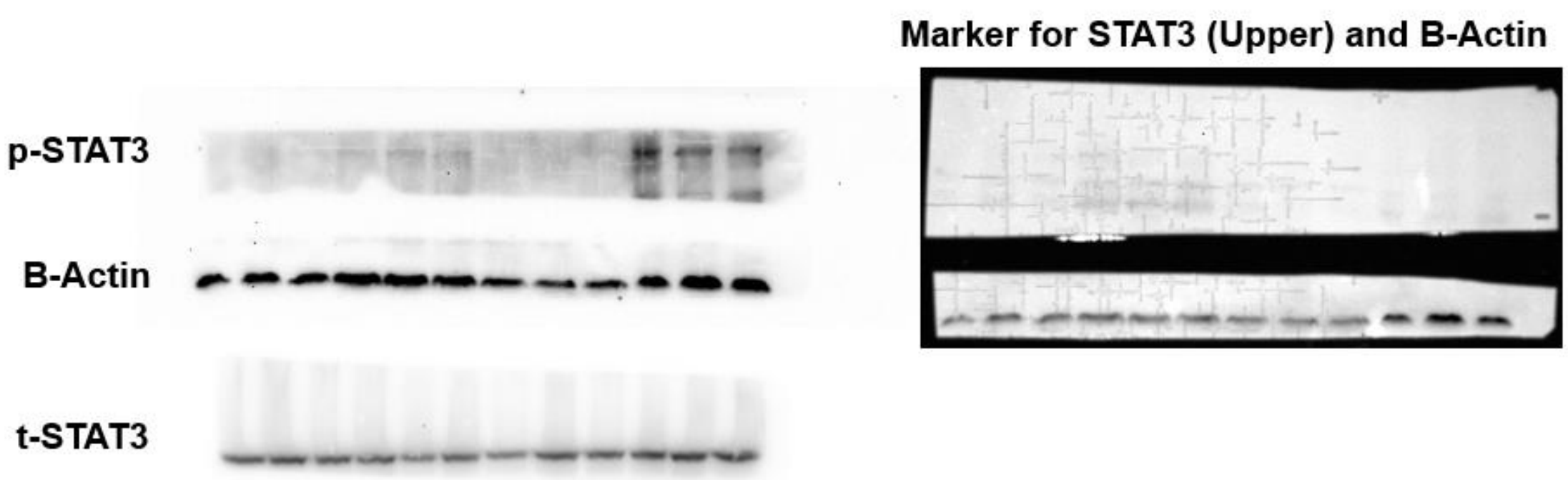
Marker for STAT3 (Upper) and B-Actin
p-STAT3
B-Actin
t-STAT3

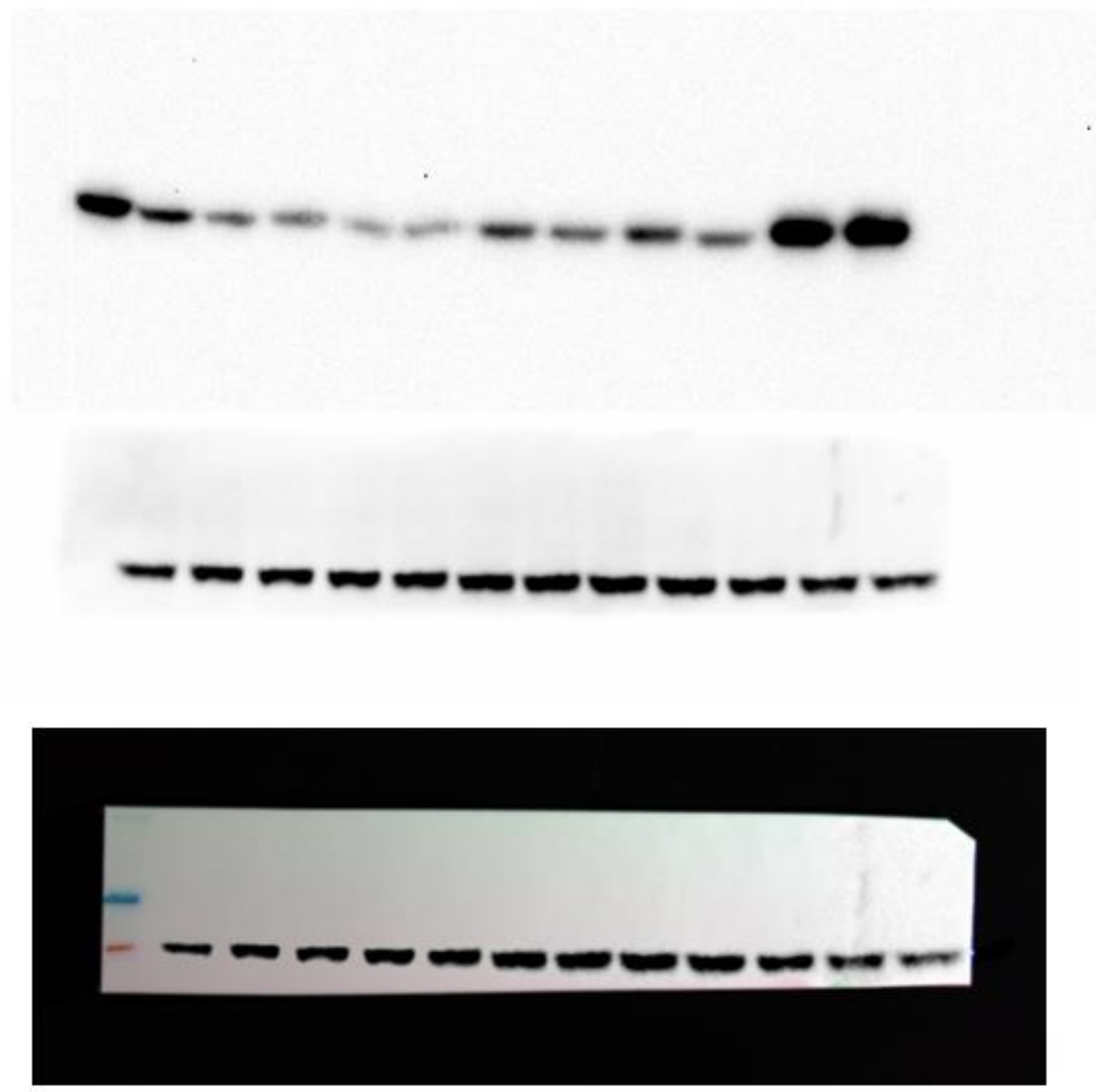
p-PKC delta
T-PKC delta
T-PKC delta with Marker

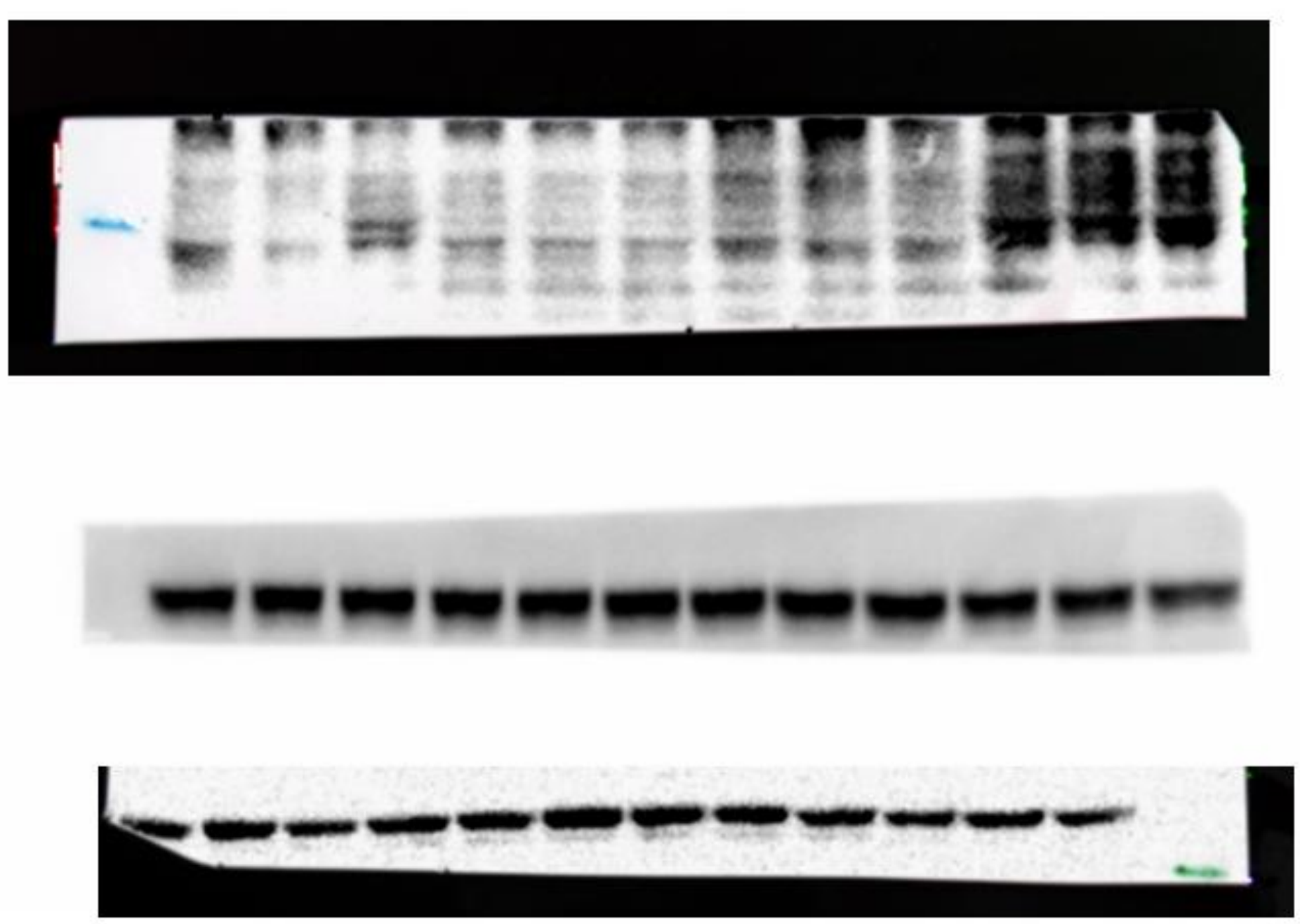
p-SMAD3/4
T-SMAD3/4
B-Actin

Supplementary Figure S1D

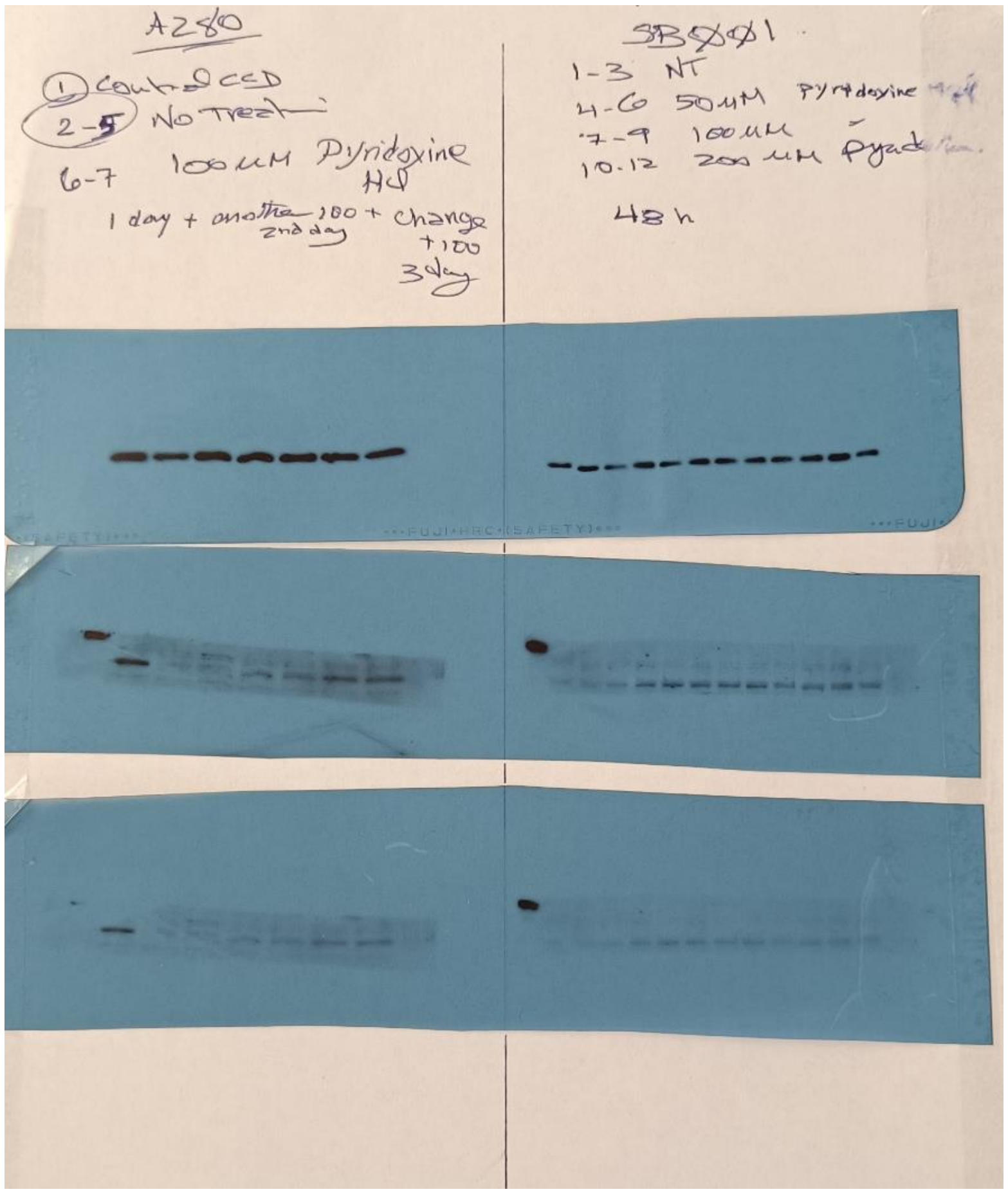

Supplementary Figure S7 Blots

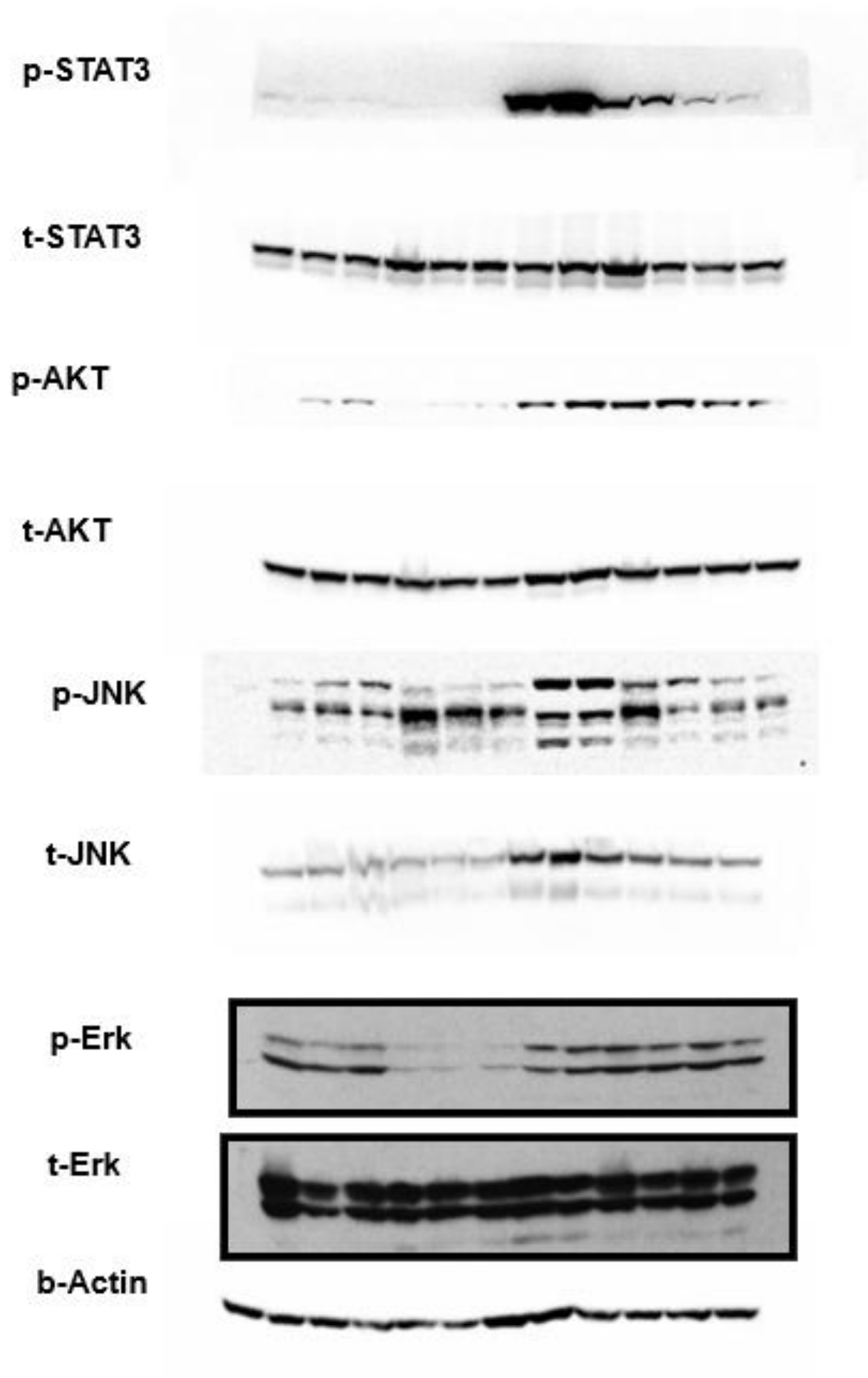

Supplement: Supplementary file 2 [file jci-136-206980-s107.pdf]
